# 3-D pillar-and-scaffold nanostructures for integrating nitrogen-vacancy doped nanodiamonds

**DOI:** 10.1039/d6ra00555a

**Published:** 2026-04-13

**Authors:** Faisal Ishaq, Sumeer Khanna, Roger Narayan, Joshua Bader, Faraz Ahmed Inam, Stefania Castelletto

**Affiliations:** a Department of Physics, Aligarh Muslim University Aligarh Uttar Pradesh 202002 India faraz.inam@gmail.com; b Department of Materials Science and Engineering, North Carolina State University Raleigh NC 27695-7907 USA; c Joint Department of Biomedical Engineering, North Carolina State University Raleigh NC 27695-7907 USA rjnaraya@ncsu.edu; d School of Engineering, RMIT University Melbourne VIC 3000 Australia stefania.castelletto@rmit.edu.au

## Abstract

Three-dimensional (3-D) scaffolds made of polymers are relevant for cell culture and sensor immobilization to convert biological signals into optical signals for detection. We present a computational study of 3-D pillar-and-scaffold nanostructures designed for embedding the negatively charged nitrogen vacancy (NV^−^) color centers in nanodiamonds, focusing on light confinement and emission enhancement. To develop optically active platforms for quantum sensing, we analyze the electric field profiles in both pillar-without-scaffold and pillar-and-scaffold dielectric structures at the zero phonon line of the NV^−^ in nanodiamonds. The results reveal that scaffold edges significantly improve field confinement by modifying the local electromagnetic environment. To further enhance resonance effects in low-index materials, a partial silver coating was applied, enabling the excitation of surface plasmon polaritons and leading to stronger field localization. Coupling of the NV^−^ center in nanodiamond with a pillar-and-scaffold structure at optimal field hotspot locations leads to a higher Purcell factor in dielectric pillar-and-scaffold and metal-coated pillar-and-scaffold structures compared to pillar-without-scaffold structures. Both dielectric and metal-coated pillar-without-scaffold structures exhibit a flat optical response with minimal emission enhancement. In comparison, dielectric and metal-coated pillar-and-scaffold structures support well-defined resonance modes and exhibit significantly higher theoretical decay rates, with the metal-coated structures showing the strongest enhancement due to plasmonic field amplification. We have fabricated 3-D pillar scaffolds with silver coating using two-photon polymerization, showing an experimental unloaded *Q*-factor of up to 17 in the spectral range of the NV^−^. These findings highlight the potential of plasmon-based pillar-and-scaffold platforms for higher-sensitivity 3-D quantum sensing using NV^−^ color centers.

## Abstract 2

Three-dimensional (3-D) scaffolds made of polymers are relevant for cell culture and sensor immobilization to convert biological signals into optical signals for detection.

## Graphical abstract

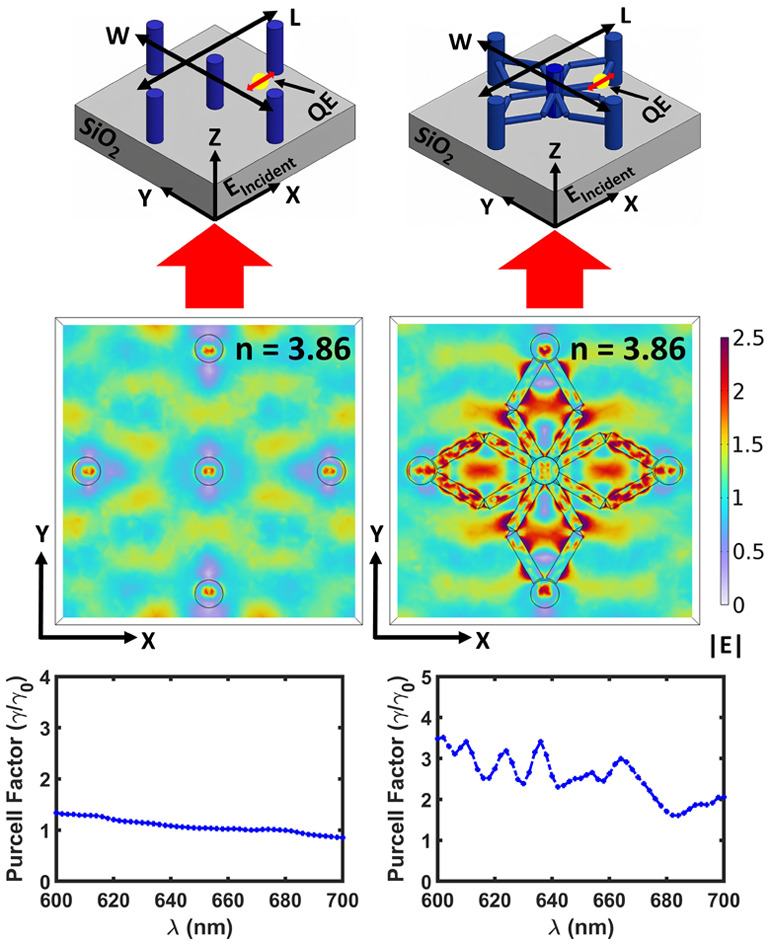

## 1 Introduction

3-D pillar-and-scaffolds are used in biosensing to better mimic the cellular environments and study the cell behavior.^1,2^ In particular, high-aspect-ratio nanostructures are suitable to deliver nutrients or drugs into cells and tissues, permit access to the intracellular environment, and can control cell behavior.^3^ Due to the 3-D pillar-and-scaffold large surface areas, they can also provide physical support to incorporate nanosensors for the detection of specific biomolecules, nutrients, or mechanical/electrical changes within the cellular environment for quantitative, real-time measurements.^4^ Nanostructures coated with a thin film layer of noble metals (silver (Ag) or gold (Au)) show specialized properties of surface plasmons because they provide the capability to focus light into deeper sub-wavelength regimes. Consequently, such 3-D nanostructures will find useful applications in various photonic devices and biosensors.^5^ The main advantages of nanosensors embedded in a 3-D plasmonic scaffold are flexibility in design with controlled shape and dimensions, purity and crystallinity of the metal layer, and the ability to concentrate plasmonic activity in areas of interest, such as the edges of horizontal scaffolds.^6,7^ Incorporating quantum sensors into plasmonic and photonic 3-D scaffolds using facile fabrication processing^8^ can further enhance their resolution and sensitivity, extending from the nanoscale to the microscale sensing volumes. Among the most studied quantum sensors, the negatively charged nitrogen vacancy (NV^−^) centers in nanodiamonds stand out due to their high biocompatibility and photostability, long spin coherence at room temperature, and optical emission centered in the visible range with a zero-phonon line (ZPL) of 637 nm.^9,10^ Nanodiamonds and diamond nanostructures with NV^−^ center are proposed for drug delivery, bioimaging, and biosensing.^11^ Therefore, NV^−^ in nanodiamonds embedded into 3-D plasmonic and photonic scaffolds can provide highly precise measurements of magnetic moments, electric fields, strain, temperature and pH with nanoscale spatial resolution^12,13^ over the large volume of the scaffold, paving the way for functional devices.^14^ Our structure's figure of merit is the enhancement of the electric field confinement and the associated enhancement of the fluorescence emission rate and brightness, based on the Purcell enhancement (*F*_P_ = *γ*/*γ*_0_), defined as the modification of the spontaneous emission rate in structured environments (*γ*) compared to the vacuum (*γ*_0_).^15^

**Fig. 1:**
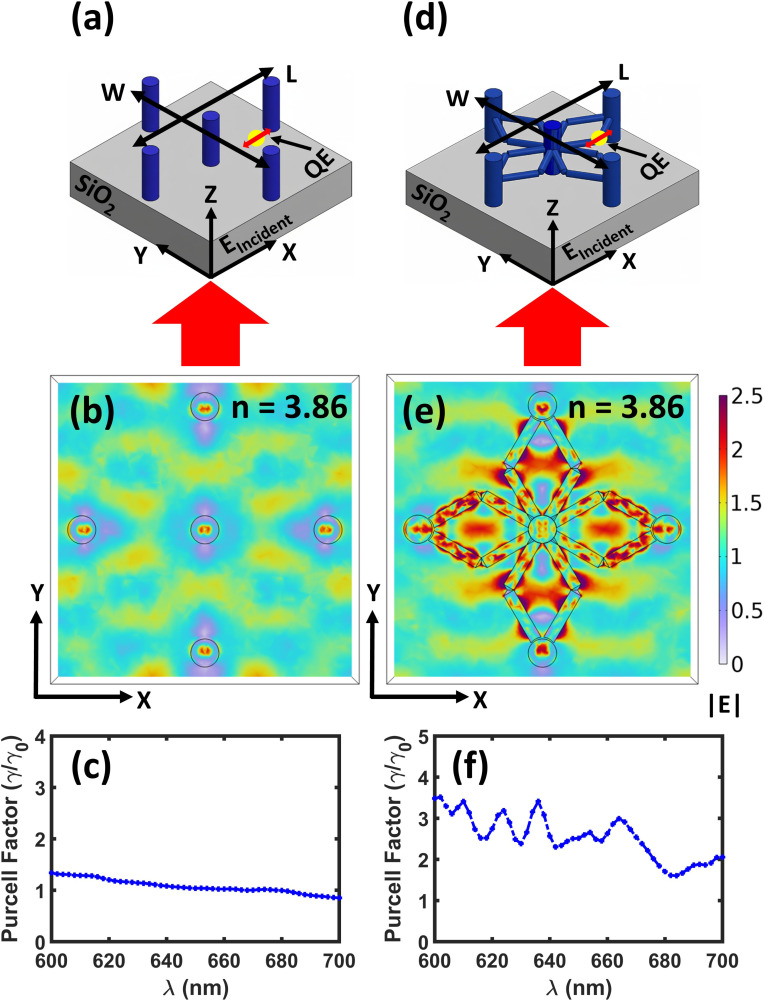
Schematic illustration of crystalline silicon (*n* = 3.86) vertical cylindrical pillars (a) without scaffolds (pillar-without-scaffold) and (d) with scaffolds (pillar-and-scaffolds), placed on a silica substrate (SiO_2_), illuminated from the bottom side by *a* plane wave with the electric field polarized along the *x*-axis. (b and e) Corresponding |**E**| profile in the *xy*-plane at the wavelength of *λ* = 637 nm. Purcell enhancement spectrum for (c) Si-based pillar-without-scaffold structure and (f) pillar-and-scaffold structure, showing resonances.

In this work, we investigate the role of 3-D nanostructured pillar-and-scaffolds to modify electromagnetic field confinement to achieve spontaneous emission enhancement. To enable a direct comparison, we analyze the electric field (|**E**|) profiles in both pillar-without-scaffold [Fig. 1(a)] and pillar-and-scaffold dielectric structures [Fig. 1(d)] at the ZPL of the NV^−^ color center in a nanodiamond. These field distributions reveal how electromagnetic energy is localized within and around the structures [Fig. 1(b and e)]. We observe that the pillar-and-scaffold structure results in significantly enhanced electric field confinement [Fig. 1(e)] compared to the pillar-without-scaffold structure [Fig. 1(b)]. Here, the scaffold joints (edges) in pillar-and-scaffold structures are not arbitrary structural elements; rather, they are deliberately engineered electromagnetic features. These joints introduce controlled geometric discontinuities and curvature variations within the geometry, which act as preferential sites for electromagnetic field localization.^15^ Under plane-wave excitation, these discontinuities promote surface charge accumulation, resulting in stronger electric fields and the formation of confined electromagnetic hotspots leading to an increased local density of optical states (LDOS) in the vicinity of the junction regions. Since the spontaneous emission rate of a quantum emitter is directly proportional to the LDOS of its surrounding photonic environment,^15^ placing an emitter near these joint regions enhances spontaneous emission *via* the Purcell effect. In addition to the localized field enhancement, the scaffold joints facilitate electromagnetic coupling between adjacent vertical pillars. This inter-pillar coupling modifies the collective resonant behavior of the structure. Therefore, the scaffold joints are deliberately engineered to function as electromagnetic coupling nodes and field-confinement centers, rather than serving merely as structural connectors within the pillar-and-scaffold system. As a result, the strongest field enhancement occurs near the scaffold edges, where pronounced optical hotspots form due to the increased local density of optical states (LDOS). To assess light–matter interaction, we compute the Purcell enhancement factor by placing a quantum emitter (QE) at identified hotspot locations in both geometries, using materials with varying refractive indices. The pillar-without-scaffold structure exhibits a flat optical response with no distinct resonances [Fig. 1(c)], resulting in negligible Purcell enhancement. In contrast, the pillar-and-scaffold structure supports resonant modes and exhibits enhanced Purcell [Fig. 1(f)]. These results demonstrate that placing the emitter at key electromagnetic hotspots, such as scaffold edges, maximizes emission enhancement. The interconnected scaffold geometry promotes stronger field confinement, thereby increasing emitter brightness and improving sensing sensitivity. Furthermore, a systematic enhancement of the Purcell factor is observed with increasing dielectric refractive index, highlighting the crucial role of refractive-index contrast in strengthening optical mode confinement and light–matter interactions. To further enhance the coupling efficiency, we investigate plasmonic effects by introducing a thin (50 nm) silver coating to both structures. In the pillar-and-scaffold configuration, the Purcell factor increases by approximately 20-fold, whereas it remains negligible in the pillar-without-scaffold case. The most significant enhancement is achieved in the silver-coated pillar-and-scaffold structures, where hybridization between plasmonic modes and dielectric resonances results in substantially improved light–matter interaction.^16,17^ Overall, these findings establish plasmon-enhanced dielectric scaffold architectures as highly tunable and efficient platforms for enhancing the performance of quantum emitters in nanoscale quantum sensing applications.

As a proof of concept, we fabricated a 3-D pillar-and-scaffold using two-photon polymerisation (2 PP) in polymers and coated the pillar-and-scaffold with Ag, showing plasmonic resonances *via* reflectance measurements at the emission wavelength of the NV^−^, providing a pathway for future integration of the quantum sensor in such scaffolds.^18,19^ The direct laser writing (DLW) process based on 2 PP was employed because it's one of the promising fabrication technologies for 3-D micro- and nano-structures^18^ with high resolution (shape precision) at comparatively faster speeds than traditional lithography methods. Secondly, DLW has been extensively utilized in recent years for the fabrication of photonic device structures for quantum sensing.^19^ The pillar-and-scaffold nanostructures coated with a thin film layer of noble metal (Ag or Au) show specialized properties of surface plasmons because they provide the capability to focus light into deeper subwavelength regimes. These 3-D nanostructures, which exhibit high sensitivity, are expected to find useful applications in various photonic devices, such as optical and biomedical sensors,^20,21^ data storage, solar energy harvesting, high-power electronics,^22–25^ and light emitters. One of the main advantages is that the design of structures can be varied on a need basis for enhanced plasmonic performance. Furthermore, these structures are unique for this application because we can control variables such as (a) shape and dimensions of structures, (b) purity and crystallinity of metal layer, and (c) ability to concentrate plasmon activity in focused areas of interest, such as the edges of horizontal scaffolds. The structures can also be coated with a thin film layer of metal alloy catalysts^26^ to deposit conductive layers of graphene or carbonized material to convert them directly into Q-carbon^20^ and nanodiamond crystallites.^21^

## 2 Methods

### 2.1 Design, theoretical and computational framework

#### 2.1.1 Design

All the simulations in this study were performed using the finite element method (FEM)-based COMSOL Multiphysics software. Detailed information is provided in the Computational methods section of the SI (Section 1). For all simulation results presented in the manuscript, a single set of lateral dimensions, *L* = *W* = 3.1 µm, with a lattice periodicity of 3.4 µm has been consistently used across all configurations, including the pillar-without-scaffold, pillar-and-scaffold, and metal-coated pillar-and-scaffold structures. This geometry was selected as the optimized structure for our study to perform the comparative analysis under identical geometric conditions to ensure clarity and consistency. We did not modify the lateral dimensions (*L* and *W*) of the structure in the simulation study. The geometry was kept fixed, and only the refractive index of the pillar-and-scaffold material was varied from polymer (*n* = 1.50) to 6H-SiC (*n* = 2.63) and crystalline silicon (*n* = 3.86) in order to systematically investigate the influence of dielectric contrast on the optical response. However, in the experimental part, we used a *L* = *W* = 40 µm structure due to limitations of the experimental setup. Therefore, exact correspondence between theory and experiments is not possible in this study. However, the experimental results will help us understand the scaffold's influence on tuning the dipole emission of embedded emitters. A detailed description of the fabrication procedure is presented in subsection 2.2 (Fabrication). In this study, both the pillar-without-scaffold and pillar-and-scaffold structures were investigated using a range of dielectric materials with varying refractive indices, including polymer, 6H-SiC, and Si. To explore plasmonic effects, a 50 nm thick Ag coating was applied to each structure. All geometries were placed on a silica (SiO_2_) substrate to simulate realistic experimental conditions.

#### 2.1.2 Theoretical framework

When an electromagnetic wave is incident on a dielectric structure, or a dielectric structure with a metallic coating, it induces spatially varying charges and current distributions within the material.^27–32^ The induced current density is calculated as*J*_*ω*_(*r*) = *iωε*_0_(*ε*_*r*_ − 1)*E*_*ω*_(*r*),where *ε*_0_ and *ε*_*r*_ are the permittivities of free space and the dielectric material (*e.g.*, polymer, SiC, or Si), respectively.^28^ The computationally obtained values of *E*_*ω*_(*r*) and *J*_*ω*_(*r*) are then used to analytically calculate the individual multipolar Mie-scattering moments; the electric and magnetic dipole moments (ED and MD), the electric and magnetic quadrupole moments (EQ and MQ) and other higher-order moments.^27,28^ The strength of these Mie scattering modes is dependent on the structure dimensions, with the higher-order modes being dominant when the structure dimensions are comparable to the excitation wavelength.^27,28,30^ We computed these moments using COMSOL simulations for both the pillar-without-scaffold and pillar-and-scaffold structures. The detailed derivations of the exact expressions for these moments are provided in ref. 27. For details about these calculations, refer to the SI (Section 2).

#### 2.1.3 Computational framework

In this study, we investigate the interaction of light with 3-D pillar-and-scaffold and pillar-without-scaffold nanostructures to evaluate their effectiveness in enhancing light–matter coupling. Two types of excitations were used: plane wave excitation to analyze the |**E**| profile and scattering efficiency, and dipole excitation to assess spontaneous emission rate enhancement and scattering efficiency near localized electromagnetic hotspots. We begin by examining the electric field distribution under plane wave illumination at 637 nm.^33^ The structure is excited using a plane-polarized electromagnetic wave (polarized along the *x*-axis), incident from the bottom side of the structure. The resulting |**E**| profiles are computed in both the *xy*- and *xz*-planes to visualize field confinement and hotspots formation around the structural features. We then calculated and identified the individual Mie-scattering moments of the scaffold, leading to the hotspot formation and thereby determining its total scattering efficiency (SE). These results are presented in the SI.

Following this, we studied the dipole excitation behavior in the scaffold by placing a QE at previously identified hotspot locations within the structure. This allows us to evaluate the local enhancement of spontaneous emission by computing the Purcell enhancement factor (*F*_P_ = *γ*/*γ*_0_). This approach enables a direct quantification of how structural geometry and material properties influence light–emitter coupling at the nanoscale. All simulations were performed using periodic boundary conditions to model light–matter interactions within an infinitely repeating periodic structure.

### 2.2 Fabrication

In this research study, an assembly of four scaffolds (horizontal pillars) and four vertical pillars are connected to a common vertical pillar, providing numerous areas of concentration for plasmonic or optical activity. Additionally, the horizontal scaffold can be assumed as a spring (suspended structure) to provide the coupled effect from structural vibration frequencies (modal effects) and surface plasmonic action of the structure. Here, a diamond-shaped structure was chosen to provide enhanced confinement of electric and magnetic fields in the horizontally placed scaffolds, in addition to vertical pillars. This effect is readily evident in the COMSOL Multiphysics modeling results, as shown in Fig. 3 and 5, of the manuscript. Secondly, this configuration provides edge tips (scaffold joints) where two arms are connected. This is an ideal location for placing NV diamond crystallites or acting as an efficient emission center for surface plasmon waves. Other geometries, such as 3-D ovals or toroids, do not consist of scaffold joints, so they will not provide a similar opportunity for integrating quantum emitters and plasmon sensing (refer to Fig. 4 and 6).

**Fig. 2:**
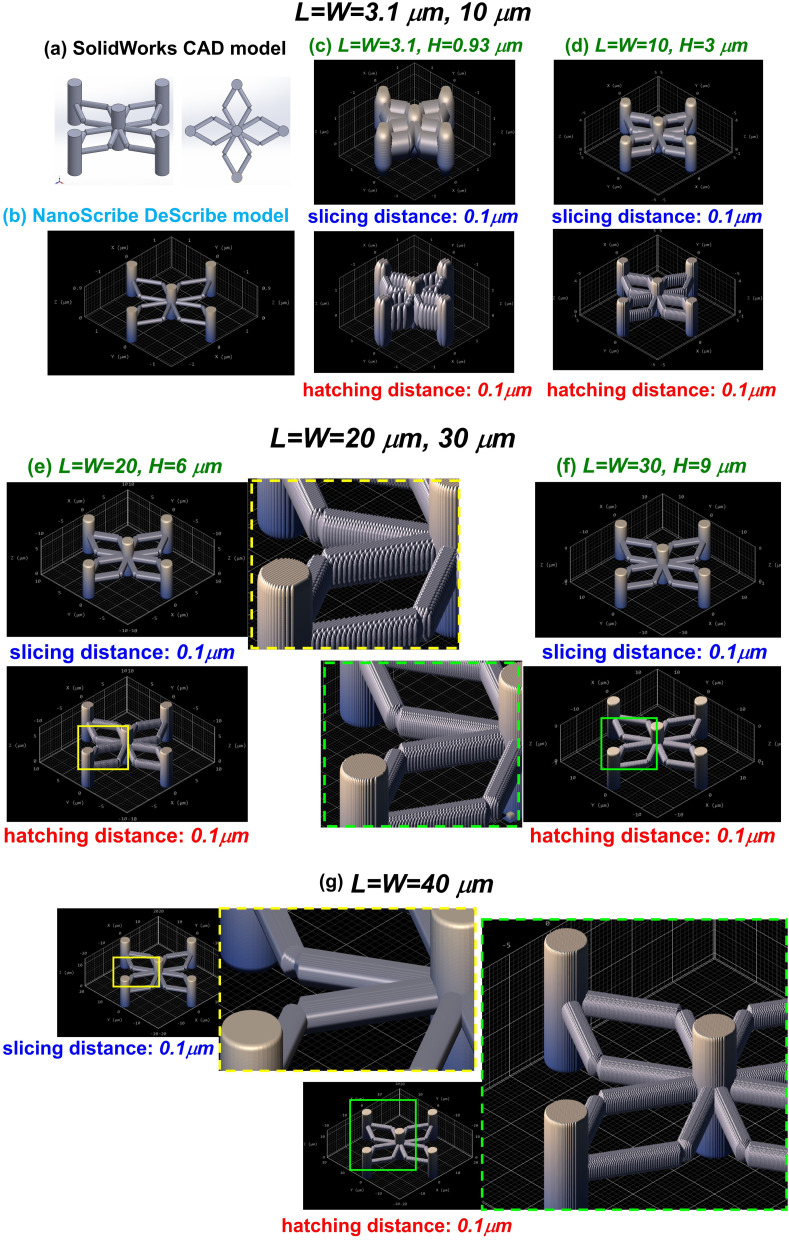
2 PP fabrication parameters for the pillar-and-scaffold structure in NanoScribe DeScribe: (a) SolidWorks CAD model; (b) NanoScribe DeScribe preview of 3-D structure after importing *.STL file; slicing distance of 0.1 µm (top) and hatching distance of 0.1 µm (bottom) for varying *L* = *W* lengths of (c) 3.1 µm, (d) 10 µm, (e) 20 µm, (f) 30 µm and (g) 40 µm.

The 2 PP process was used here because of its ease in fabricating complex 3-D structures, such as the pillar-and-scaffold geometry with high precision at the nanometer scale and unequalled shape accuracy. The focused-electron-beam-induced (FEBiD) process^34^ could also be employed; however, its precision for complex 3-D structures and throughput are limited on a larger scale. The fabrication sequence [Fig. 2(g)] for the 3-D structures consisted of first modeling the design concept in the SolidWorks [Fig. 2(a)] software platform to generate the CAD file, which was then converted into a stereolithography (*.STL) file format. The DLW lithography process based on the 2 PP process employed by the NanoScribe photonic professional GT2 requires a *.JOB file to get started with printing. The latter is generated using the NanoScribe DeScribe software [Fig. 2(b)] by inputting the *.STL file which consists of the parameters of CAD structure. An array of 10 × 10 (100) features were created over a footprint area of 2 µm × 2 µm. The advantage of using DeScribe software is that you can set the slicing distance (horizontal layers) and hatching distance (parallel lines) for one 3-D structure, which essentially governs the precision (edge sharpness and fineness of dimensions) of features and the time required for fabrication using the DLW process. The optimization of these parameters [Fig. 2(g)] was one of the primary goals for this research study to enhance the mechanical stability of structures.

We started by setting the dimensions (*L* = *W* = 3.1 µm) of the structures [Fig. 2(c)], the same as those used in COMSOL Multiphysics to fabricate the structures with identical dimensions. However, the NanoScribe lithography tool is limited or restrained in fabricating structures to about their sub-wavelength (*λ*/2 = 780/2) feature size of 390 nm. As per specifications provided by the manufacturer, it can print features of size up to 100–200 nm. The curved edges of pillar-and-scaffold features are in the range of 10–20 nm for *L* = *W* = 3.1 µm [Fig. 2(c)]. Secondly, when this model was loaded in DeScribe, the edge features completely bulge out. The dimensions of the features were thus affected. Therefore, to reduce this intolerance, we increased the dimensions of *L* and *W* parameters of the pillar-and-scaffold structures sequentially from 3.1 µm [Fig. 2(c)] → 10 µm [Fig. 2(d)] → 20 µm [Fig. 2(e)] → 30 µm [Fig. 2(f)] → 40 µm [Fig. 2(g)]. The corresponding decrease in the bulge of structures and accuracy of structures can be readily observed by this enhancement. After these fabrication parameters were set for the model, the *.JOB file generated by DeScribe was then loaded to the NanoScribe NanoWrite software for fabricating the 3-D structure. 63× objective is employed for writing finer structures using the IP Dip-2 photo resin. After fabrication, the structures were coated with a thin film layer of 50 nm of Ag using the Orion PVD sputter tool available at the NC State Nanofabrication Facility (NNF). The Ag deposition was done using Orion magnetron DC sputtering tool equipped with a 2″ Ag target set to power of 50 W, voltage of 344 V and current of 144 mA. The substrate was set to a rotation of 50 rpm during the run. The plasma was strike in the chamber by flowing Ar at 30 sccm, pressure of 30 mT and power of 30 W. PVD chamber base pressure was maintained to 1.5 × 10^−7^ T and the deposition pressure was 5 mT. Ag coating of approximately 500 Å (50 nm) was obtained during a deposition run time of 6 minutes. To obtain uniform Ag coating on the scaffold structure, the Ag deposition was done at varying angles of ∼10°, ∼25° and ∼45° by employing small plastic shims of varying heights on substrate holder. The Si substrate with 3-D patterned structures were mounted at the edge of this shim touching sample holder. See the images in the SI (Fig. S7) for your reference. There is a major aberration in feature sharpness and dimensions for model with dimensions of *L* = *W* = 3.1 µm [Fig. 2(c)], which is reduced as the length of structure along the *X* and *Y* planar axes are increased to *L* = *W* = 10 µm [Fig. 2(d)]. However, there is still abnormality in feature shapes. The zoom-in views of scaffold curved edges for *L* = *W* = 20 µm [Fig. 2(e)] and 30 µm [Fig. 2(f)] are shown adjacent to the structures, respectively bearing yellow (top) and green (bottom) dotted borders. The accuracy of feature shapes is still deviant, but they are reduced as the dimensions are increased. Finally, we can observe from the zoom-in views of scaffold curved edges adjacent to the structures for *L* = *W* = 40 µm that the anomaly in feature sharpness is reduced.

As shown in Fig. 2, the resolution of 3-D printed structures is determined by the slicing and hatching distances used in the NanoScribe DeScribe software to prepare the printing job file. This, in turn, is dependent upon the wavelength (*λ* = 780 nm) of the laser employed. The resolution of the minimum feature size is approximately sub-wavelength dimensions, which is 390 nm. However, according to the manufacturer, a minimum feature size of 100 nm and shape accuracy of 200 nm in the *XY* plane can be achieved. As per the Comsol modeling, we had optimized the dimensions of scaffold structures to *L* = *W* = 3.1 µm as the minimum feature size. The dimensions of curved (circular) features of the scaffold surface were found to be ∼10–50 nm. These lengths are below the NanoScribe's current resolution limit. Accordingly, you can see in the feature preview option while preparing the printing files in DeScribe, as shown in Fig. 2(c) and (d), there is a bulge in the scaffold features, and the shape accuracy is comprised even at the lowest possible slicing and hatching distances of 0.1 µm (100 nm). To improve the shape precision of the scaffold, the size of the structure was enlarged to *L* = *W* = 40 µm from their initial values of 10 µm to 20 µm to 30 µm. The hatching and slicing distances were kept to their minimum values of 0.1 µm (100 nm) for all cases. As you can see in Fig. 2(g), the surface features for *L* = *W* = 40 µm are very smooth as compared to others, and thus, the shape accuracy for the scaffold structure was maintained.

## 3 Results and discussion

### 3.1 Dielectric pillar-and-scaffold structure

**Fig. 3:**
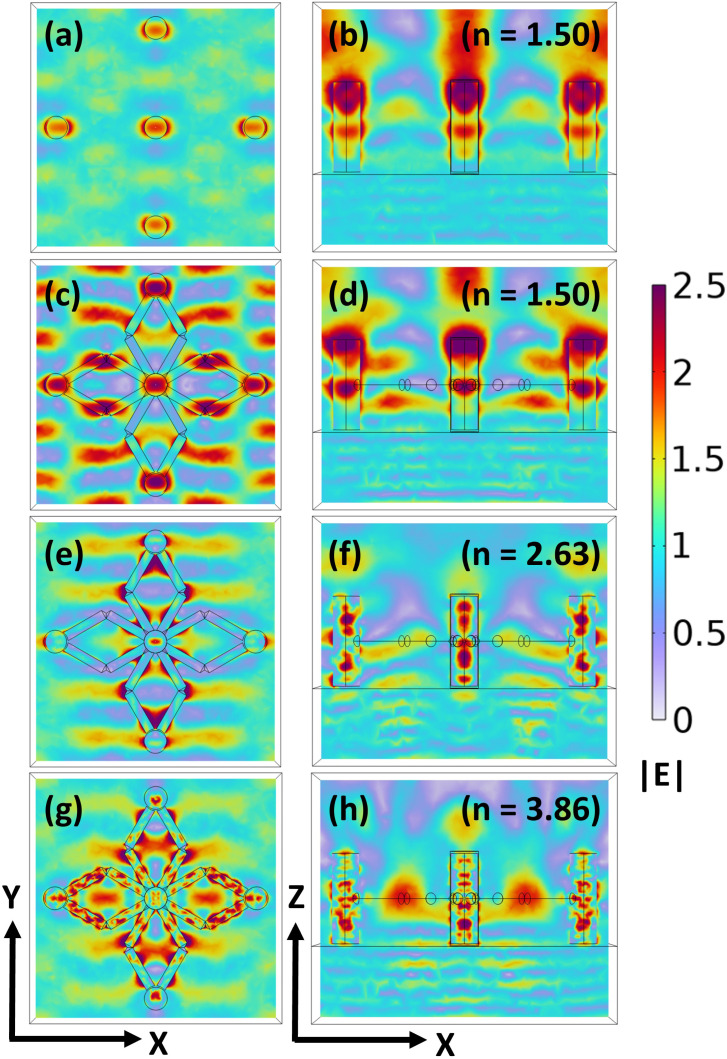
(a and b) |**E**| profiles in the *xy*- and *xz*-planes for the pillar-without-scaffold structure composed of a polymer of refractive index (*n* = 1.50), placed on a silica substrate (SiO_2_), illuminated by *a* plane wave incident from the bottom side. (c, e and g) |**E**| profiles in the *xy*-plane for the pillar-and-scaffold structure composed of a polymer (*n* = 1.50), 6H-silicon carbide (6H-SiC, *n* = 2.63), and crystalline silicon (Si, *n* = 3.86), respectively, placed on a SiO_2_ substrate, illuminated by *a* plane wave incident from the bottom side. (d, f and h) Corresponding |**E**| profiles in the *xz*-plane for the pillar-and-scaffold structures composed of a polymer, SiC, and Si, respectively.

We begin by analyzing the |**E**| profiles for a pillar-without-scaffold structure composed of a polymer and placed on an SiO_2_ substrate, and it is excited from the substrate side using *a* plane wave polarized along the *x*-axis, allowing us to quantify the baseline electromagnetic response in the absence of scaffolds. The resulting |**E**| profiles in the *xy*-plane [Fig. 3(a)] and *xz*-plane [Fig. 3(b)] reveal that the field is concentrated near the top surfaces of the vertical pillars and the central regions. For comparison, we extend our analysis to the polymer-based pillar-and-scaffold structure under the same excitation conditions, enabling a direct assessment of the scaffolds' influence on electromagnetic field confinement. The corresponding |**E**| profiles in both *xy*-plane [Fig. 3(c)] and *xz*-plane [Fig. 3(d)] show a notable increase in the number of high-field regions, particularly around the scaffold edges, as well as enhanced confinement along the vertical pillars. The presence of scaffolds introduces additional geometric features that support localized field enhancement, leading to stronger light confinement within the structure. These localized field enhancements play a crucial role in quantum sensing applications, particularly when using QEs such as NV^−^ color centers in nanodiamonds. The additional hotspots around the scaffold regions provide multiple high-field zones that can serve as optimal sites for QE placement. The pillar-and-scaffold structure offers improved sensing performance through enhanced Purcell factors and increased spatial resolution in detecting local electromagnetic field perturbations.

The material of the pillar-and-scaffold structure was then changed to high-indexed SiC, and the same analysis was performed. The results, illustrated in [Fig. 3(e)] and [Fig. 3(f)] for the |**E**| profiles, reveal a greater number of hotspots as compared to the polymer case, which indicates higher electric field confinement due to the SiC higher refractive index. Owing to this property, the material supports stronger optical confinement, thereby enhancing light–matter interaction at the emission wavelength. Finally, we replace the material of the pillar-and-scaffold structure with Si and perform the same analysis. The results, presented in [Fig. 3(g)] and [Fig. 3(h)], reveal a greater number of electric field hotspots compared to the previous cases. Our results demonstrate that introducing horizontal scaffolds into the vertical pillar lattice significantly enhances electromagnetic field confinement by generating additional hotspots. We evaluated the SE behavior of the horizontal pillar-and-scaffold structure and observed that the scaffold structure resulted in the strong excitation of multipolar Mie-scattering modes within the scaffold. These results are presented in the SI (Section 4). Tuning the refractive index of the pillar-and-scaffold system from a polymer to SiC and ultimately to Si systematically strengthens the field confinement and promotes more efficient light–matter interaction (Fig. 3).

In the studied structures, the primary factor influencing light confinement is the local electromagnetic field enhancement induced by geometric discontinuities and refractive index contrast. In particular, sharp curvature regions and junctions between interconnected elements promote charge accumulation and localized field enhancement, thereby increasing the local density of optical states (LDOS) and enhancing light–matter interaction. Another factor affecting the light confinement is the formation of four diamond features in the scaffold structure. This is better than toroids and rectangular bars because it provides two scaffold joints for a single diamond feature. Circular features of the scaffold are preferable to rectangular bars, as they provide uniform light confinement and dissipate surface plasmon polariton energy from the scaffold. Additionally, scaffold joints are preferred sites for placement of the NV diamond defects in the structure, which will provide efficient coupling of light from defect centers along with the surface plasmon polaritons. Therefore, coupling a QE, such as an NV^−^ center in a nanodiamond, to localized electromagnetic hotspots can significantly enhance its interaction with external fields. When positioned at these bright, field-enhanced regions, the NV^−^ center experiences stronger coupling to the local electromagnetic environment, directly contributing to enhanced sensitivity in quantum sensing applications.

**Fig. 4:**
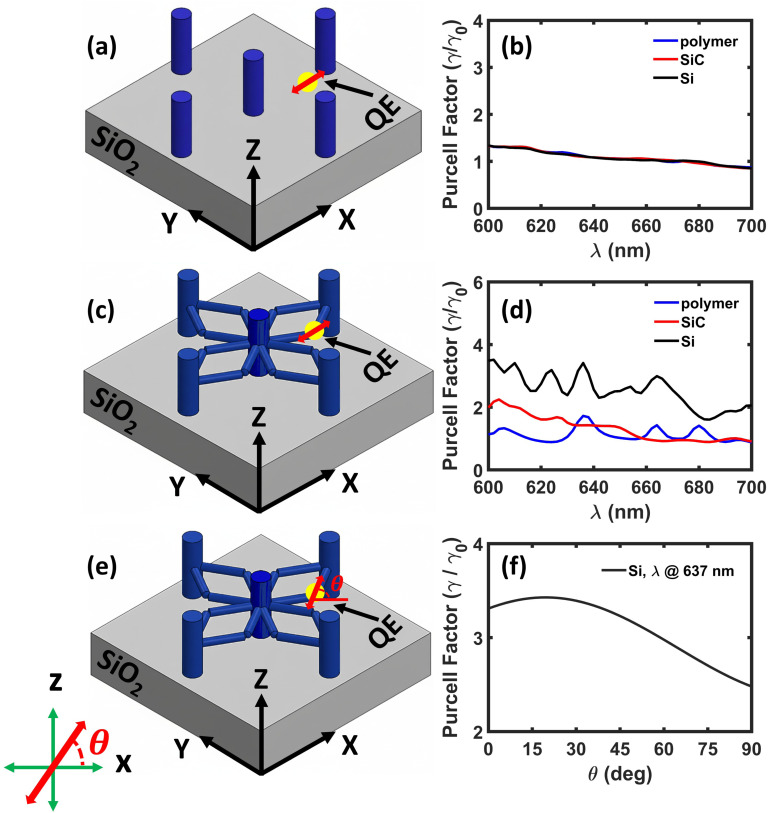
Schematic illustrations of a quantum emitter (QE) oriented parallel to the *x*-axis and coupled to (a) the pillar-without-scaffold structure and (c) to the pillar-and-scaffold structure, both placed on a SiO_2_ substrate. Corresponding Purcell enhancement spectra for the (b) pillar-without-scaffold structure and (d) the pillar-and-scaffold structure, with the same dielectric materials: polymer, SiC, and Si. (e) Schematic illustration of a QE with an angular dipole orientation coupled to a pillar-and-scaffold structure composed of silicon (*n* = 3.86) and being placed on a SiO_2_ substrate. (f) Decay rate behaviour as a function of dipole orientation (*λ* = 637 nm) for the Si pillar-and-scaffold structure.

To evaluate the influence of identified electromagnetic hotspot locations on a QE performance, we investigate the dipolar emission of NV^−^ in nanodiamond embedded within both pillar-without-scaffold and pillar-and-scaffold structures by computing the Purcell enhancement. This comparison highlights the role of the scaffold in modifying the emitter's radiative properties. In the pillar-without-scaffold configuration, the emitter is placed directly on the top surface of the silica substrate, as shown in [Fig. 4(a)]. In comparison, for the pillar-and-scaffold structure, the QE is positioned at one of the scaffold arm junctions, an identified field hotspot illustrated in [Fig. 4(c)]. To ensure a correct comparison, the QE in both cases shares the same in-plane (*x*, *y*) coordinates. However, the *z*-coordinate differs slightly due to experimental constraints, as the QE in the pillar-without-scaffold structure must be placed on a solid surface and cannot be suspended in air. The analysis is carried out for parallel dipole orientations (lying along the *x*-axis) in both geometries. We perform this study using different dielectric materials, such as polymer, SiC, and Si, to assess the influence of refractive index on Purcell enhancement in both structures. For the pillar-without-scaffold structure, the Purcell enhancement spectra exhibit a flat optical response across all materials, indicating the absence of well-defined resonant modes [Fig. 4(b)]. Consequently, the spontaneous emission enhancement remains negligible throughout the examined wavelength range, suggesting weak coupling between the QE and the photonic environment due to limited field confinement. In comparison, the pillar-and-scaffold structure exhibits distinct resonance peaks in the Purcell spectra for all three dielectric materials [Fig. 4(d)]. These resonances lead to a noticeable increase in emission rates, with enhancement factors that scale with the material's refractive index. Specifically, we observe a 2-fold enhancement for polymer, a 2-fold enhancement for SiC, and up to a 4-fold enhancement for silicon. These results highlight the critical role of scaffold geometry and refractive index in enabling stronger light–matter interaction by increasing field confinement, which in turn enhances the LDOS.

To investigate the influence of dipole orientation on the spontaneous emission, the Purcell factor is evaluated as a function of dipole orientation from 0° (along *x* – axis) to 90° (along *z* – axis) for the Si pillar-and-scaffold structure (refer to Fig. 4(e) and (f)). The results indicate that when the dipole is oriented parallel to the *x*-axis, *i.e.*, along the plane of the horizontal scaffolds, the Purcell factor reaches an enhancement of more than three times. This enhancement arises because the dipole radiation effectively couples to the resonant modes of the scaffold when its orientation aligns with the dominant electric field distribution within the geometry. As the dipole orientation gradually rotates toward the perpendicular direction (along the *z*-axis), the Purcell enhancement decreases. In this configuration, the dipole radiation is less efficiently coupled to the structural resonant modes, leading to weaker light–matter interaction and reduced emission enhancement. However, the observed Purcell enhancement is well above the pillar-without-scaffold case.

Here in this study our main aim is to study/evaluate the role of the scaffold structure in enhancing electromagnetic field confinement and QE's spontaneous emission. Therefore, we focused on evaluating the QE's emission performance at the hotspot locations within the scaffold structure. However, for completeness, we have evaluated the QE emission performance at locations within the vertical pillars. For QE embedded within the pillar, the scaffold's influence was observed to be minimal. These results are presented in the SI (Section 5).

### 3.2 Silver-coated dielectric pillar-and-scaffold structure

**Fig. 5:**
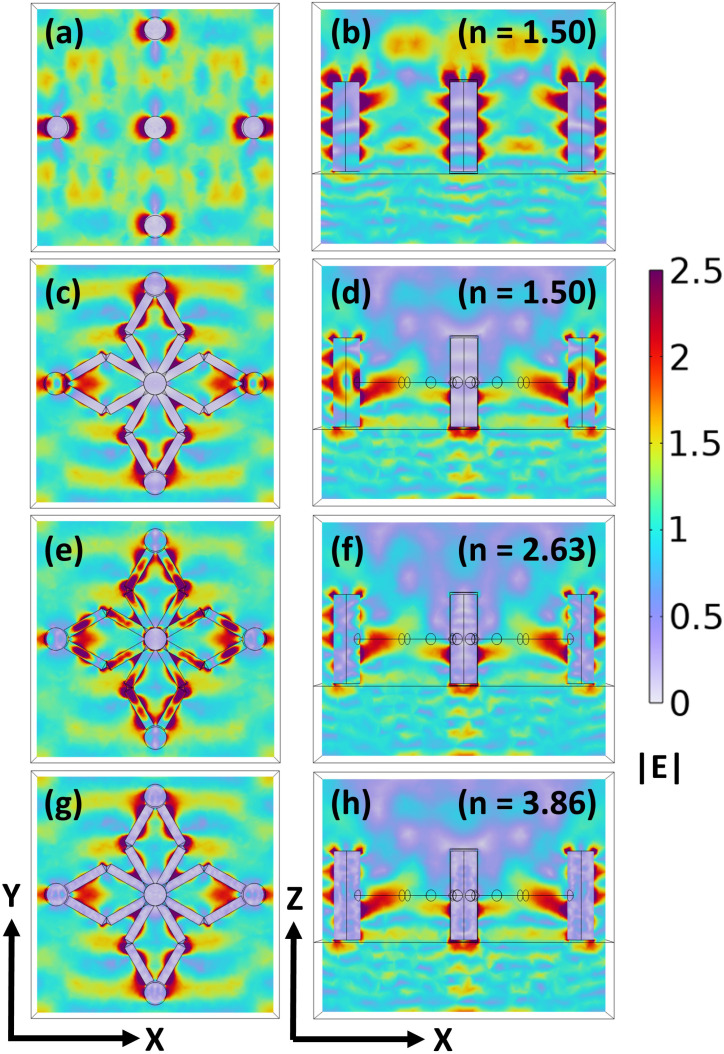
(a and b) |**E**| profiles in the *xy*- and *xz*-planes for the silver-coated pillar-without-scaffold structure composed of polymer (*n* = 1.50), placed on a SiO_2_ substrate and excited by *a* plane wave incident from the bottom side. (c, e and g) |**E**| profiles in the *xy*-plane for the silver-coated pillar-and-scaffold structure composed of a polymer (*n* = 1.50), SiC (*n* = 2.63), and Si (*n* = 3.86), respectively, placed on a SiO_2_ and illuminated by *a* plane wave incident from the bottom side. (d, f and h) Corresponding |**E**| profiles in the *xz*-plane for the silver-coated pillar-and-scaffold structures composed of a polymer, SiC, and Si, respectively.

We then investigate the impact of a 50 nm thin silver coating on both pillar-without-scaffold and pillar-and-scaffold dielectric structures composed of various dielectric materials, including polymer, SiC, and silicon. The analysis is conducted under plane-wave excitation conditions, with particular attention paid to the spatial distribution of the |**E**| profiles in the *xy*- and *xz*-planes. We begin by examining the silver-coated pillar-without-scaffold structure composed of polymer, illuminated by *a* plane wave polarized along the *x*-axis incident from the bottom of the structure. The resulting |**E**| profiles [Fig. 5(a and b)] indicate strong field confinement at the surfaces of the pillars, accompanied by the emergence of localized hotspots. These surface hotspots arise due to the surface plasmon polariton (SPP) excitation at the metal–dielectric interface.^35^ To explore the impact of scaffold integration, we extend the analysis to a silver-coated pillar-and-scaffold structure made from the same polymer. The corresponding |**E**| profiles [Fig. 5(c and d)] show field enhancement not only at the pillar surfaces but also along the scaffold edges. The presence of additional intense hotspots around the scaffold elements signifies enhanced SPP-mediated field localization due to the plasmonic surface and connectivity introduced by the scaffolds. Subsequently, we investigate material-dependent behavior by replacing the polymer with SiC in the scaffolded structure while maintaining the silver coating thickness. As shown in [Fig. 5(e and f)], the SiC-based configuration exhibits a greater number of brighter and more localized hotspots at the metal–dielectric interfaces due to stronger electromagnetic confinement and more efficient plasmonic coupling. Finally, we consider a Si-based scaffold structure with an identical silver coating. The resulting electric field profiles [Fig. 5(g and h)] resemble those observed in the polymer-based counterpart, with field localization primarily occurring at the pillar-and-scaffold surfaces. Despite silicon's higher refractive index compared to polymer, the hotspot intensity remains similar, suggesting that material dispersion and plasmonic mode overlap play significant roles in determining the overall field enhancement.

**Fig. 6:**
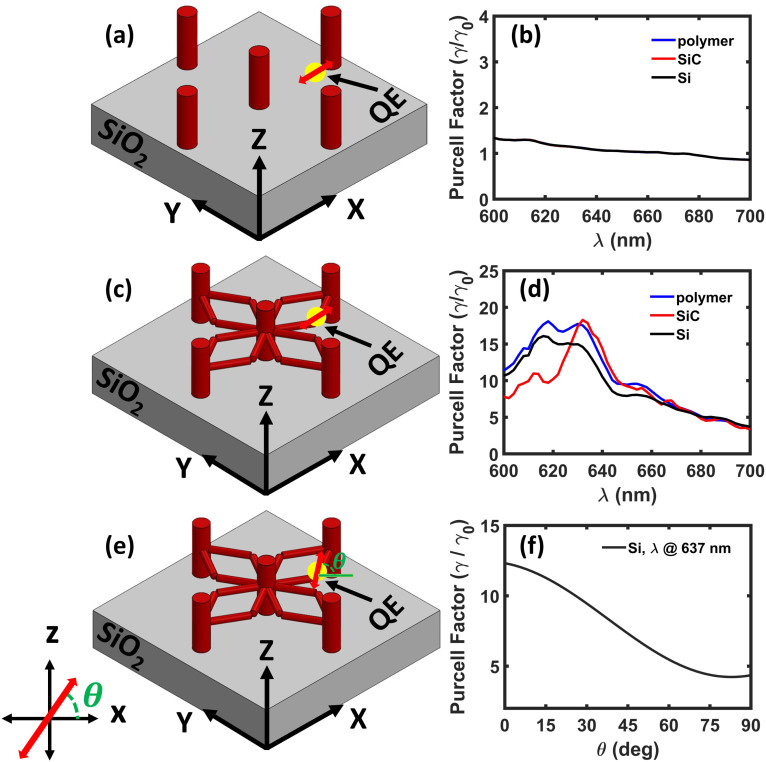
Schematic illustrations of a quantum emitter (QE) oriented parallel to the *x*-axis and coupled to (a) a silver-coated pillar-without-scaffold structure and (d) a silver-coated pillar-and-scaffold structure with different dielectric materials: polymer, SiC, and Si. Both structures are placed on a SiO_2_ substrate. Corresponding Purcell enhancement spectra for (b) the silver-coated pillar-without-scaffold structure and (d) the silver-coated pillar-and-scaffold structure with the same dielectric materials: polymer, SiC, and Si. (e) Schematic illustration of a QE with an angular dipole orientation coupled to a metal-coated pillar-and-scaffold structure composed of silicon (*n* = 3.86) and placed on a SiO_2_ substrate. (f) Decay rate behaviour as a function of dipole orientation (*λ* = 637 nm) for the Si pillar-and-scaffold structure.

We further investigate the dipolar emission of NV^−^ in a nanodiamond embedded in a dielectric pillar-and-scaffold structure coated with a 50 nm silver layer to evaluate the impact of plasmonic effects on spontaneous emission through Purcell enhancement. Similar to the dielectric case, the QE is placed directly on the top surface of the silica substrate in the pillar-without-scaffold configuration [Fig. 6(a)]. As before, in the pillar-and-scaffold structure, the QE is positioned at a scaffold arm junction, as shown in [Fig. 6(c)]. The analysis is carried out for parallel dipole orientations (lying along the *x*-axis) in both geometries, *i.e.*, pillar-without-scaffold and pillar-and-scaffold structures composed of different dielectric materials: polymer, SiC, and Si, each coated with a thin silver layer. In the silver-coated pillar-without-scaffold structures, the Purcell enhancement spectra remain flat across all materials, showing no distinct resonant modes [Fig. 6(b)]. As a result, the spontaneous emission enhancement is negligible, indicating weak light–matter coupling and a lack of significant plasmonic interaction in the absence of structural features that support field localization. In comparison, the silver-coated pillar-and-scaffold structures exhibit clear resonance peaks in the Purcell spectra for all dielectric materials considered [Fig. 6(d)]. These resonances lead to substantial enhancement in the emission rate, with Purcell factors scaling with the refractive index of the dielectric. Specifically, we observe approximately a 20-fold enhancement for both polymer and SiC, and up to a 17-fold enhancement for silicon. These results signify the dual role of plasmonic coupling and scaffold geometry in amplifying light–matter interaction by enhancing field confinement and increasing the LDOS.

Further, we perform a similar analysis as in the dielectric case to investigate the influence of dipole orientation on the spontaneous emission of a quantum emitter coupled to a metal-coated pillar-and-scaffold structure. The Purcell factor is evaluated as a function of dipole orientation from 0° to 90° to understand how the emitter–structure coupling depends on the alignment of the dipole moment with the scaffold geometry. This analysis is performed for the metal-coated pillar-and-scaffold structure composed of Si, as illustrated in [Fig. 6(e)]. The corresponding Purcell enhancement spectrum is shown in [Fig. 6(f)]. In this case, the metallic coating introduces plasmonic modes that interact with the dipole emission. When the dipole is oriented parallel to the *x*-axis, the Purcell factor exhibits a strong enhancement of approximately 13-fold. This enhancement arises due to efficient coupling between the dipole radiation and the plasmonic modes supported by the metal-coated structure. As the dipole orientation gradually shifts toward the perpendicular direction, the Purcell enhancement decreases because the dipole radiation couples less efficiently with the resonant modes of the structure. Nevertheless, the overall trend remains consistent with the dielectric case: the maximum Purcell enhancement occurs for dipole orientations parallel to the scaffold plane, while the minimum enhancement is observed for perpendicular dipole orientations.

The dipole orientation of the quantum emitter strongly influences its coupling to the scaffold's electromagnetic modes. Since realistic emitters may adopt arbitrary orientations, the overall emission behaviour can be interpreted as an effective average of these configurations.

### 3.3 Fabricated silver-coated polymer pillar-and-scaffolds

**Fig. 7:**
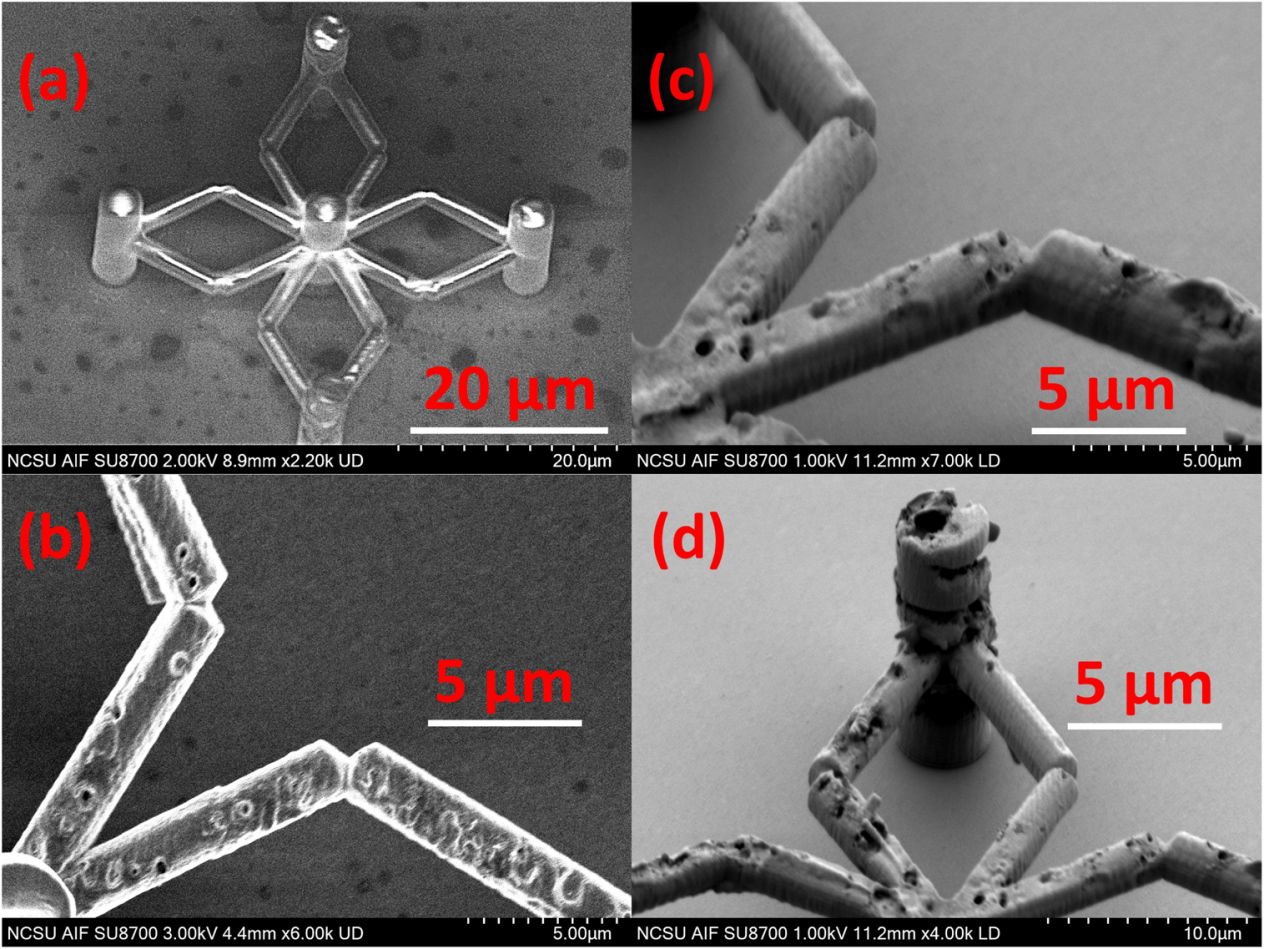
Fabricated 3-D structures (*L* = *W* = 40 µm) coated with a thin film plasmonic Ag layer of 50 nm thickness, optical (a and b) and HR-SEM images (c and d), respectively.

The 3-D fabricated structures were imaged using Pixel LINK (PL-A661) optical microscope [Fig. 7(a and b)] and analyzed for by a higher resolution scanning electron microscope (HR-SEM) (Hitachi SU8700 HR-SEM) shown in Fig. 7(c and d). As it can be inferred from the HR-SEM images, the scaffold edge sharpness and dimensions match well to the CAD model input to the 2 PP DLW system. The thin shiny coating on top of the scaffold edges is Ag metallic layer (≈50 nm) deposited by a Physical Vapor Deposition (PVD) sputter system. The fine surface finish of the metallic layer on top of the fabricated structure shows that subsequent processing of the fabricated structures is feasible, and the features don't break. Furthermore, the 2 PP system is highly reproducible, and we can fabricate large array of features at once in comparatively less time as compared to traditional photo- and e-beam lithography techniques. An array of 20 × 20 features were fabricated in approximately 8 hours.

**Fig. 8:**
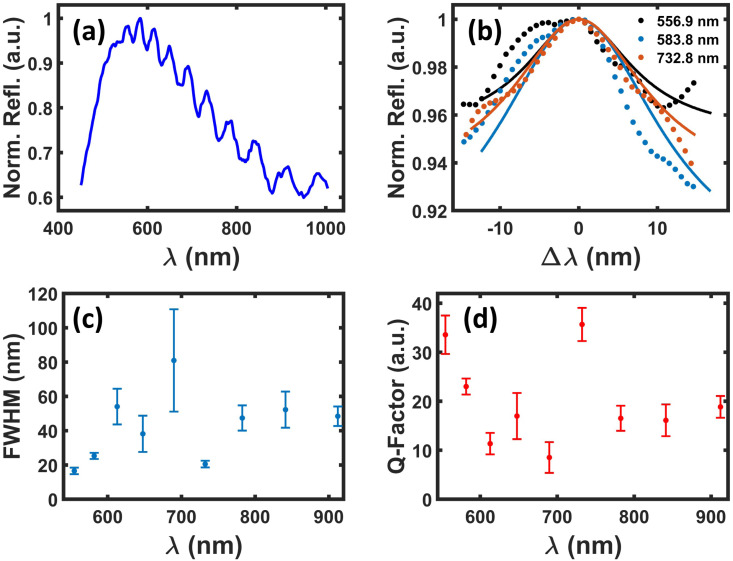
(a) Measured Normalized reflectance (Norm. Refl. (a.u.)) spectrum from the same 3-D-pillars-scaffold. (b) Three narrowest observed optical resonances for wavelengths (*λ* = 556.9 nm, *λ* = 583.8 nm, *λ* = 732.8 nm) with measured data points (dotted) and an applied single Lorentzian fit (solid lines). (c) Overview of all extracted FWHM values for all individual optical resonances in relation to the observed wavelength, where the vertical error bar illustrates fit uncertainties. (d) Overview of the exhibited *Q*-factors for all optical resonances in relation to the observed wavelength, where the vertical error bar represents the propagated uncertainty from the FWHM determination.

The optical characteristic response of the scaffold is provided *via* reflectance measurements, focusing on the scaffold edges, using a CRAIC Apollo Microspectrophotometer. As shown in [Fig. 8(a)], we observe a noticeable reflectance increase between 454.8 nm and 526.8 nm, with distinct resonances ranging from 554.67 nm until 912.16 nm. We identify similar shapes considering the three narrowest optical resonances situated at 554.67 nm, 581.64 nm and 732.28 nm shown within the inset of [Fig. 8(b)]. By applying a single Lorentzian fit, we can determine the narrowest full width half maximum (FWHM) values of (16.52 ± 1.93) nm, (25.28 ± 1.78) nm and (20.52 ± 1.95) nm, respectively. Furthermore, broader resonances at 612.82 nm, 689.76 nm and 841.21 nm provide a fitted FWHM of (54.02 ± 10.43) nm, (80.91 ± 29.85) nm and (52.23 ± 10.52) nm, as shown in [Fig. 8(c)]. Finally, we determine the *Q*-factors (directly linked to Purcell enhancement *F*_p_ ∝ *Q*/*V*, *V* mode volume) from the structure for each individual resonance as shown within [Fig. 8(d)] and observe maximum values of *Q* = 35.67 ± 3.39 and *Q* = 33.56 ± 3.92 at 554.67 nm and 732.28 nm, respectively. Furthermore, *Q*-factors of *Q* = 11.34 ± 2.19, *Q* = 16.97 ± 4.7 and *Q* = 8.52 ± 3.14 can be identified at 612.82 nm, 647.69 nm and 689.76 nm, respectively. The FWHMs and resonance centres *λ*_*i*_ were extracted from the fitted Lorentzian distributions, where individual uncertainties (*σ*_FWHM_ and *σ*_*λi*_) are obtained from the square roots of the diagonal elements from the covariance matrix returned by the nonlinear least-squares fitting technique. The presented *Q*-factor (*Q* = *λ*_*i*_/FWHM) takes both fitted parameter into account (*λ*_*i*_ and *FWHM*) where the inferred *Q*-uncertainties were determined by:
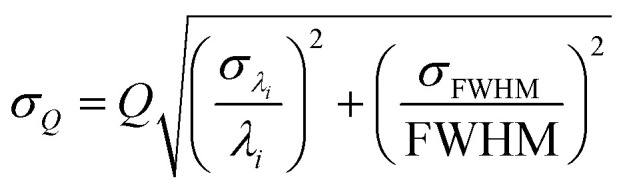
assuming uncorrelated fit parameters.

We have characterized five scaffolds, all providing the same resonances, here we show the highest *Q*-factors achieved.

Considering the previously determined FWHMs, the optical resonance situated at 647.69 nm appears to partially overlap with the PL emission wavelength of an NV^−^ at 637 nm, which could lead to an enhancement in the spontaneous emission rate, subject to future studies. By tuning the scaffold sizes, specific resonances could be tailored to not only NV in nanodiamonds, rather to other spin defects exhibiting optical detected magnetic (ODMR) resonances, needed for quantum sensing, and easier to integrate in scaffolds such as QEs in hexagonal boron nitride^36^ with closer proximity to the plasmonic resonances, QEs in smaller silicon carbide nanoparticles^37^ or directly induced by direct femto-second laser writing in polymers.^38^

## 4 Conclusion

This study highlights the importance of scaffold geometry and material choice in enhancing light–matter interaction for quantum sensing applications. Compared to pillar-without-scaffold structures, pillar-and-scaffold structures significantly improve field confinement and support optical resonances, resulting in a 4-fold increase in Purcell enhancement. Introducing a silver coating further amplifies these effects, yielding up to a 20-fold enhancement due to plasmonic coupling. In comparison, pillar-without-scaffold structures exhibit flat optical responses and almost negligible emission enhancement, even with metal coating. Preliminary fabrication of plasmonic 3-D-pillar scaffold based on polymers provides clear resonance modes corresponding to QEs such as NV^−^. These results demonstrate that pillar-and-scaffold architectures are effective in boosting the performance of NV^−^ QEs and could be applied to other QEs based such as hexagonal boron nitrides flakes and carbon-related defects emitting at ≈590 nm with up to 90% optical detected magnetic resonance contrast,^39^ offering a robust and scalable platform for high-sensitivity 3-D-quantum sensing.

## Author contributions

Conceptualization and methodology: S. C., R. N., F. A. I., S. K., F. I.; fabrication: S. K.; experimental data acquisition: J. B., S. K.; visualization and interpretation: J. B., S. C., S. K., F. I.; modeling: F. A. I., F. I., S. K.; curation, visualization, and investigation: J. B., S. C., F. A. I., F. I., S. K.; supervision: S. C., F. A. I., R. N.; writing original manuscript draft: S. C., F. I., S. K.; reviewing and editing: S. C., F. I., S. K. All authors contributed to writing and commenting the final manuscript.

## Conflicts of interest

The authors declare that they have no known competing financial interests or personal relationships that could have appeared to influence the work reported in this paper.

## Supplementary Material

RA-016-D6RA00555A-s001

## Data Availability

The data underlying this study are not publicly available due to ongoing related research and intellectual property considerations. The data are available from the corresponding authors upon reasonable request. Supplementary information (SI): additional details on computational methodology, theoretical framework for scattering efficiency (SE), emission dynamics of a quantum emitter (QE), SE of pillar-without-scaffold, pillar-and-scaffold, and metal-coated pillar-and-scaffold structures and the analysis of dipole coupling with pillar-and-scaffold and pillar-without-scaffold configurations, are provided in the SI. Fig. S1 provides the details of the computational methodology used in this work. Fig. S2 shows the simulation results for the SE of pillar-without-scaffold, pillar-and-scaffold, and metal-coated pillar-and-scaffold structures. Fig. S3–S6 present the simulation results for both pillar-without-scaffold and pillar-and-scaffold configurations, where the dipole is positioned 20 nm below the top surface inside the central pillar. The study includes dielectric structures (Fig. S3 and S4) and Ag-coated structures (Fig. S5 and S6), for two dipole orientations: parallel (‖) to the *x*-axis and perpendicular (⊥) to the *xy*-plane (i.e., along the *z*-axis). See DOI: https://doi.org/10.1039/d6ra00555a.
