# Supplementary material for: 3-D pillar-and-scaffold nanostructures for integrating nitrogen-vacancy doped nanodiamonds
Source: RSC Adv. 2026 Apr 13;16(21):19397–411. doi: 10.1039/d6ra00555a (PMC13075127; doi:10.1039/d6ra00555a)
Supplement: RA-016-D6RA00555A-s001 [file RA-016-D6RA00555A-s001.pdf]

Supplementary Information (SI) for RSC Advances.
This journal is © The Royal Society of Chemistry 2026

RSC Advances

SUPPLEMENTARY INFORMATION

3-D pillar-and-scaffold nanostructures for integrating nitrogen-vacancy doped nanodiamonds

Faisal Ishaq[a], Sumeer Khanna[b], Roger Narayan*[c], Joshua Bader[d], Faraz Ahmed Inam[a], Stefania Castelletto[d]

1 Computational methods

The COMSOL model of the performed simulations is shown in Figure S1. The first row of Figure S1 presents the structural parameters and overall details of the vertical pillars-without-scaffold structure, while the second row shows those of the pillars-and-scaffold structure. First, unit cell geometries for both pillar-without-scaffold and pillar-and-scaffold lattice structures were initially designed in Dassault Systemes SolidWorks and Autodesk Inventor as 3-D CAD models and subsequently imported into COMSOL Multiphysics for numerical simulation.

To ensure accurate dimensional scaling, the imported geometries, originally defined in meters, were uniformly scaled by a factor of 0.533×10^{-5} to convert them into the appropriate microscale dimensions. In FigureS1(a), the top view of the pillar-without-scaffold structure is shown. Here, L and W denote the overall dimensions of the structure along the x- and y-directions, respectively, each measuring $3.1\,\mu$m from one end to the other. The pillar diameter (D) is 320 nm, and the periodicity of the structure is 3.4 μm. A nanodiamond sphere with a radius of 50 nm is modeled, containing a negatively charged nitrogen-vacancy center (NV^-) that serves as a quantum emitter (QE). Figure S1(b), representing the front view of the structure. The pillar height H is 1066 nm, and the pillars are placed on a silica (SiO_2) substrate with a height of 867 nm. The top domain consists of air with a height of 1933 nm, giving a combined total height of 2800 nm, and the position of the quantum emitter (QE) is indicated by an arrow. For better visualization, a 3D view of the COMSOL geometry is presented in [Figure S1(c)]. Similarly, Figure S1(d) shows the top view of the pillar-and-scaffold structure with the same parameters and overall dimensions as the pillar-without-scaffold case. In this configuration, additional horizontal pillars are attached to the vertical pillars. Each horizontal pillar has a diameter of d = 160 nm and a height of h = 906 nm as shown in Figure S1(d). The horizontal pillars are symmetrically connected to the vertical ones, extending along both the x- and y-directions. These connections interlink the vertical pillars, resulting in a three-dimensional scaffold-like structure that provides enhanced electromagnetic field confinement. A quantum emitter (QE) is placed at one of the scaffold joints, as indicated in the Figure The QE is modeled as a nanodiamond sphere with a radius of 50 nm containing a nitrogen-vacancy (NV^-) center. In this simulation, the quantum emitter (QE) is modeled as an oscillating point dipole with a unit dipole moment $\vec{p}$ and a specified orientation. The orientation of the dipole can be controlled by adjusting the direction of $\vec{p}$. For the parallel dipole orientation, $\vec{p}$ is aligned along the x-axis or y-axis, while the perpendicular orientation is obtained by setting $\vec{p}$ along the z-axis. Figure S1(e) shows the front view of the pillar-and-scaffold structure placed on the same silica substrate, as discussed for the pillar-without-scaffold case, and also the 3D view of the same model is shown in Figure S1(f). The refractive index profiles for the different geometry components in COMSOL were assigned based on standard data sources. The values for silica (SiO_2) and air were selected from COMSOL's built-in material library, with silica fixed at $n = 1.45$. For the polymer, a user-defined (blank) material was created with a constant refractive index of $n = 1.5$. The 6H-silicon carbide (6H-SiC) dispersion data were taken from Wang et al., 2013[1], and the crystalline silicon (Si) data were sourced from Green, 2008[2]. The silver (Ag) optical constants were adopted from the well-established data by Johnson and Christy, 1972[3]. For the nanodiamond, refractive index data were taken from Phillip and Taft, 1964[4].

[a] Department of Physics, Aligarh Muslim University, Aligarh, Uttar Pradesh, 202002, India. E-mail: faraz.inam@gmail.com
[b] Department of Materials Science and Engineering, North Carolina State University, Raleigh, NC, 27695-7907, USA.
[c] Joint Department of Biomedical Engineering, North Carolina State University, Raleigh, NC, 27695-7907, USA. E-mail: rjnaraya@ncsu.edu
[d] School of Engineering, RMIT University, Melbourne, VIC 3000, Australia. E-mail: stefania.castelletto@rmit.edu.au

2 Theoretical framework for Scattering Efficiency

The final expressions used for calculating the Mie-scattering modes, as implemented in COMSOL, are given below.

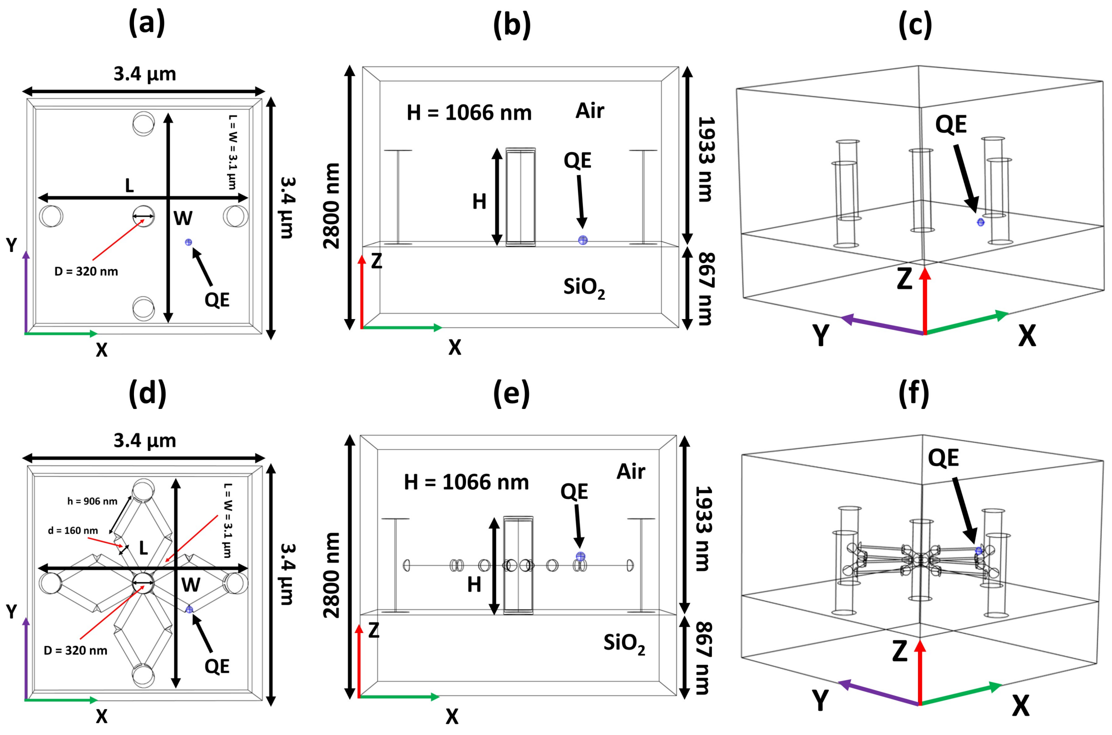

Figure S1. Schematic representation of the COMSOL model geometry: (a–c) top view (xy-plane), front view (xz-plane), and 3D view of vertical pillars-without-scaffold, showing dimensions, parameters, and material sections, and position of a quantum emitter (QE); (d–f) corresponding top view (xy-plane), front view (xz-plane), and 3D view for the pillar-and-scaffold structure.

$$\text{ED:}\quad p_\alpha = -\frac{1}{i\omega}\left[\int d^3\mathbf{r} J^\omega_\alpha\, j_0(kr) + \frac{k^2}{2}\int d^3\mathbf{r}\left(3(\mathbf{r}\cdot\mathbf{J}_\omega)r_\alpha - r^2 J^\omega_\alpha\right)\frac{j_2(kr)}{(kr)^2}\right],$$

$$\text{MD:}\quad m_\alpha = \frac{3}{2}\int d^3\mathbf{r}\,(\mathbf{r}\times\mathbf{J}_\omega)_\alpha \frac{j_1(kr)}{kr},$$

$$\text{EQ:}\quad Q^e_{\alpha\beta} = -\frac{3}{i\omega}\left[\int d^3\mathbf{r}\left(3(r_\beta J^\omega_\alpha + r_\alpha J^\omega_\beta) - 2(\mathbf{r}\cdot\mathbf{J}_\omega)\delta_{\alpha\beta}\right)\frac{j_1(kr)}{kr} + 2k^2\int d^3\mathbf{r}\left(5r_\alpha r_\beta(\mathbf{r}\cdot\mathbf{J}_\omega) - (r_\alpha J_\beta + r_\beta J_\alpha)r^2 - r^2(\mathbf{r}\cdot\mathbf{J}_\omega)\delta_{\alpha\beta}\right)\frac{j_3(kr)}{(kr)^3}\right],$$

$$\text{MQ:}\quad Q^m_{\alpha\beta} = 15\int d^3\mathbf{r}\left(r_\alpha(\mathbf{r}\times\mathbf{J}_\omega)_\beta + r_\beta(\mathbf{r}\times\mathbf{J}_\omega)_\alpha\right)\frac{j_2(kr)}{(kr)^2}. \quad (1)$$

The integration in Eq. 1 is evaluated over the combined domain of the pillars-and-scaffolds. These moments are further used to calculate the total scattering efficiency (SE), denoted as $C^{\text{total}}_{\text{sca}}$, which is obtained as the sum of contributions from various multipole moments, including C^{p}_{sca} (electric dipole), C^{m}_{sca} (magnetic dipole), $C^{Q_e}_{\text{sca}}$ (electric quadrupole), $C^{Q_m}_{\text{sca}}$ (magnetic quadrupole), and higher-order terms. It is determined by normalizing the overall far-field scattered power to the incident wave's energy flux on the scatterer[5].

$$C^{\text{total}}_{\text{sca}} = C^{p}_{\text{sca}} + C^{m}_{\text{sca}} + C^{Q_e}_{\text{sca}} + C^{Q_m}_{\text{sca}} \quad (2)$$

$$C^{\text{total}}_{\text{sca}} = \frac{k^4}{6\pi\varepsilon_0^2|E_{\text{inc}}|^2}\left\{\sum\left(|p_\alpha|^2 + \left|\frac{m_\alpha}{c}\right|^2\right)\right\} + \frac{1}{120}\sum\left(|kQ^e_{\alpha\beta}|^2 + \left|\frac{kQ^m_{\alpha\beta}}{c}\right|^2\right), \quad (3)$$

where p_α and m_α are the electric and magnetic dipole moments, with $Q^e_{\alpha\beta}$ and $Q^m_{\alpha\beta}$ being the corresponding quadrupole moments. $|E_{\text{inc}}|$ is the amplitude of the incident electric field, k is the wave vector, and c is the speed of light.

The resonant Mie scattering modes supported by the structure play a crucial role in tailoring the local electromagnetic environment, which is fundamentally governed by the local density of optical states (LDOS) and can be expressed as[6,7]:

$$\rho(\omega, r) = \sum_{k,\sigma}\left|\hat{d}\cdot\mathbf{E}_{k,\sigma}(r)\right|^2 \delta\left(\omega - \omega_{k,\sigma}\right). \quad (4)$$

Here, $\hat{d}$ denotes the unit vector representing the orientation of the transition dipole moment, while ω corresponds to the transition frequency. The summation extends over all possible wavevec-

tors (k) and polarizations (σ). The term $\mathbf{E}$ represents the total electric field at the emitter position, arising from both the field directly radiated by the dipole and the fields reflected and scattered back from the surrounding environment[7]. It governs the radiation properties of a dipole emitter by determining the available photonic modes for emission[8,9]. It is strongly influenced by the shape, size, and material composition of the surrounding structure, which modify the electromagnetic environment and thus the emission dynamics[9].

3 Emission dynamics of a quantum emitter (QE)

The emission dynamics of a quantum emitter are strongly influenced by its local electromagnetic environment, which depends on factors such as the shape, size, and material of the surrounding structure. This effect is quantified by the Purcell factor (F_p). Engineering spontaneous emission by tailoring the emitter's environment is a key strategy in various quantum optical technologies, including cavity quantum electrodynamics[10], nanophotonics[11], and quantum communication[12]. The Purcell factor (F_p) depends on the local density of optical states (LDOS), which can be tailored by modifying the local environment of the quantum emitter. Consequently, the spontaneous emission rate (SER) can be significantly enhanced. [13]. An accurate evaluation of the Purcell factor for a quantum emitter requires a quantum mechanical treatment. For a two-level quantum emitter system, the SER (decay rate, Γ) can be derived using perturbation theory as follows[14]:

$$\Gamma(\mathbf{r},\omega) = \frac{2\mu_0\omega^2}{\hbar}|\mathbf{p}|^2\,\mathrm{Im}\,[\hat{\mathbf{p}}\cdot\mathbf{G}(\mathbf{r},\mathbf{r},\omega)\hat{\mathbf{p}}]\,, \tag{5}$$

where $\mathbf{p}$ is the transition dipole moment located at position $\mathbf{r}$ and radiating with angular frequency ω. In the above equation, Green's function $\mathbf{G}$ represents the local electromagnetic environment, and $\hat{\mathbf{p}} = \mathbf{p}/|\mathbf{p}|$ denotes the unit vector along the dipole orientation.

In free space, the Green's function $\mathbf{G}_0$ gives:

$$\mathrm{Im}\,[\hat{\mathbf{p}}\cdot\mathbf{G}_0(\mathbf{r},\mathbf{r},\omega)\hat{\mathbf{p}}] = \frac{\omega}{6\pi c},$$

where c is the speed of light. Therefore, the decay rate in free space becomes:

$$\Gamma_0(\omega) = \frac{\omega^3|\mathbf{p}|^2}{3\pi\hbar\varepsilon_0 c^3}.$$

By normalizing the decay rate in the medium with respect to free space, we get:

$$\frac{\Gamma(\mathbf{r},\omega)}{\Gamma_0(\omega)} = \frac{6\pi c}{\omega}\,\mathrm{Im}\,[\hat{\mathbf{p}}\cdot\mathbf{G}(\mathbf{r},\mathbf{r},\omega)\hat{\mathbf{p}}]\,. \tag{6}$$

The above discussion is based on the quantum mechanical description of the emitter. Classically, the time-averaged power radiated by an oscillating dipole $\mathbf{p}$ at frequency ω into its surroundings can be expressed as:

$$P(\mathbf{r},\omega) = \frac{\mu_0\omega^3}{2}|\mathbf{p}|^2\,\mathrm{Im}\,[\hat{\mathbf{p}}\mathbf{G}(\mathbf{r},\mathbf{r},\omega)\hat{\mathbf{p}}]\,.$$

When normalized to the power radiated in free space, $P_0(\omega)$, we obtain:

$$\frac{P(\mathbf{r},\omega)}{P_0(\omega)} = \frac{6\pi c}{\omega}\,\mathrm{Im}\,[\hat{\mathbf{p}}\cdot\mathbf{G}(\mathbf{r},\mathbf{r},\omega)\hat{\mathbf{p}}]\,. \tag{7}$$

From Eqs. (2) and (3), the ratio obtained from these two approaches, both of which describe the spontaneous emission rate (SER) or Purcell factor enhancement, is identical. Using this equivalence, we evaluated the Purcell factor in our study by directly calculating the emitter's radiated power when placed in different environments. Specifically, if the power emitted by the dipole at one of the scaffold joints is denoted by γ, and the power radiated by the same emitter in vacuum is γ_0, then the Purcell factor is determined as:

$$F_p = \frac{\gamma}{\gamma_0} \tag{8}$$

4 Scattering efficiency of pillar-without-scaffold, pillar-and-scaffold, and metal-coated pillar-and-scaffold Structures

To understand how the structural geometry influences the optical response of the system, we calculate the scattering efficiency (SE) spectra for three configurations: a pillar-without-scaffold, a pillar-and-scaffold, and a metal-coated pillar-and-scaffold geometry, all composed of Si with lateral dimensions of $L = W = 3.1\,\mu$m. The structures are excited by a plane wave incident from the bottom side with the electric field polarized along the x-axis.

For the pillar-without-scaffold structure, illustrated in Figure S2(a), the scattering efficiency spectrum shown in Figure S2(b) exhibits a relatively weak optical response. This behavior arises because the excitation wavelength is shorter than the separation between adjacent pillars, resulting in limited electromagnetic coupling between them. Consequently, the excitation of multipolar resonances is suppressed, leading to a nearly featureless scattering spectrum. In contrast, the pillar-and-scaffold structure shown in Figure S2(c) introduces horizontal connections between the vertical pillars, which significantly modify the optical response. The presence of these scaffolds enhances electromagnetic coupling between adjacent pillars. As a result, the incident wavelength interacts more efficiently with the geometry, enabling the excitation of distinct multipolar resonances. This behavior is reflected in the scattering efficiency spectrum in Figure S2(d), where clear resonant peaks corresponding to electric quadrupole (EQ) and magnetic dipole (MD) modes are observed. Furthermore, when the pillar-and–scaffold structure is coated with a thin metallic layer of 50 nm, as shown in Figure S2(e), the optical response is further enhanced due to the excitation of plasmonic modes supported by the metal coating. The corresponding scattering efficiency spectrum in Figure S2(f) reveals sharper and more pronounced resonances compared to the purely dielectric structure. In this case, the electric quadrupole (EQ) and magnetic dipole (MD) modes are strongly enhanced by plasmonic coupling, thereby increasing the electromagnetic field confinement around the structural features. These results demonstrate that the introduction of scaffold connections and plasmonic coatings plays a crucial role in tailoring the resonant behavior of the system.

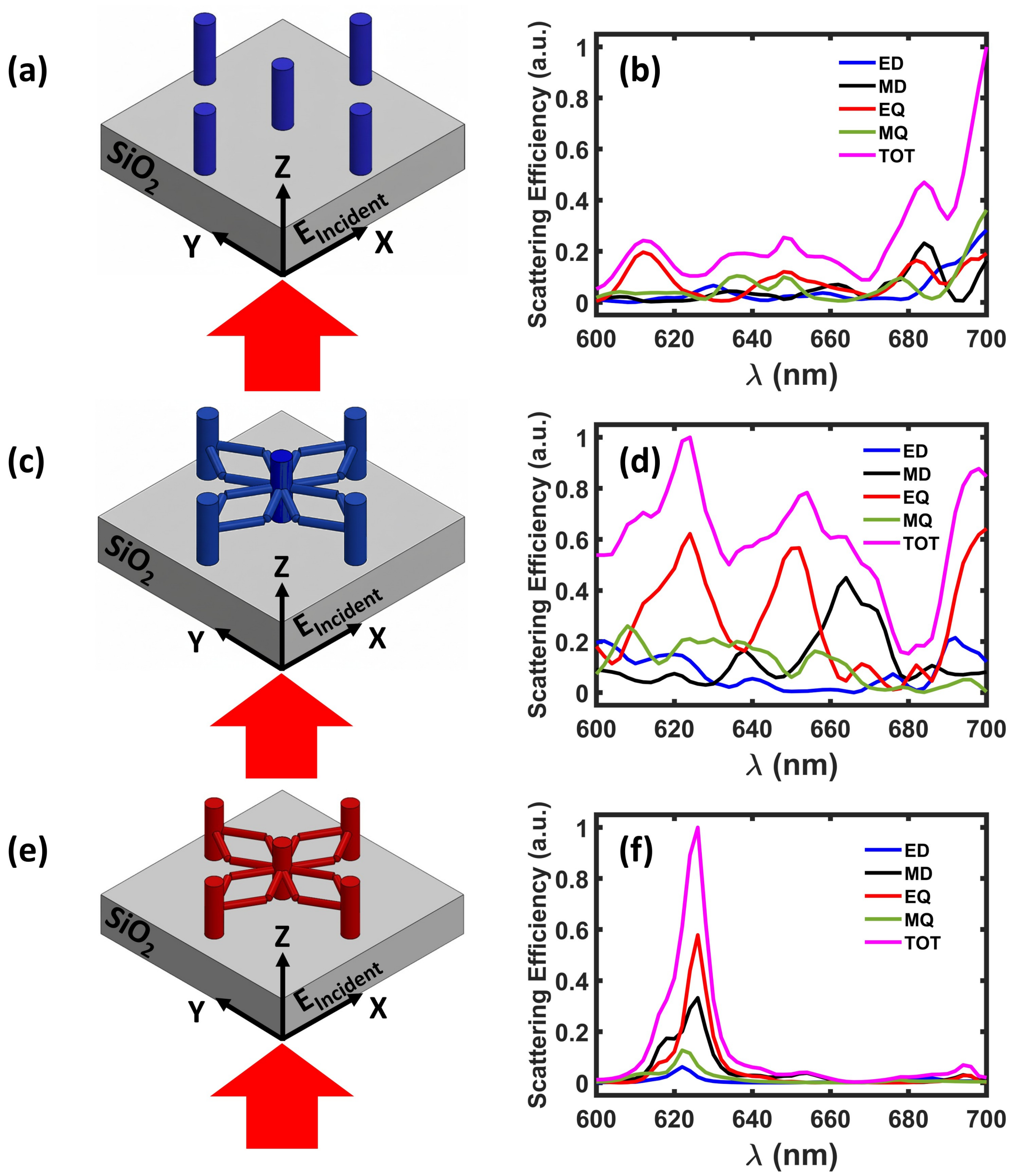

Figure S2. (a) Schematic illustration of a pillar-without-scaffold structure composed of Si placed on a silica substrate, excited by a plane wave incident from the bottom side. (b) Corresponding scattering efficiency spectrum. (c) Schematic illustration of a pillar-and-scaffold structure composed of Si placed on a silica substrate, excited by a plane wave incident from the bottom side. (d) Corresponding scattering efficiency spectrum. (e) Schematic illustration of a metal-coated pillar-and-scaffold structure composed of Si placed on a silica substrate, excited by a plane wave incident from the bottom side. (f) Corresponding scattering efficiency spectrum.

5 Analysis of Dipole Coupling with pillar-without-scaffold and Pillar-and-Scaffold Structures

5.1 Dielectric Pillar-and-Scaffold structure

Here, we discuss the coupling behavior of a negatively charged nitrogen vacancy (NV^-) center in a nanodiamond quantum emitter (QE) integrated with two types of dielectric structures: a pillar-without-scaffold and a pillar-and-scaffold structure. Both are composed of polymer and placed on a silica substrate. The QE is located inside the central vertical pillar, positioned 20 nm below the top surface, as shown in [Figure S3(a,d)]. The position of the dipole is indicated at the top of the schematics, located within the small view of the pillar.

We begin by analyzing the pillar-without-scaffold structure [Figure S3(a)], which is excited by a dipole oriented parallel to the x-axis. The corresponding scattering efficiency spectrum [Figure S3(b)] reveals the excitation of electric quadrupole (EQ) and magnetic quadrupole (MQ) modes, indicating the presence of higher-order resonances. These modes give rise to the Purcell enhancement of approximately 5-fold [Figure S3(c)]. Subsequently, we perform a similar analysis for the pillar-and-scaffold structure, as shown in [Figure S3(d)]. The scattering efficiency spectrum exhibited a comparable mode pattern to that observed in the pillar-without-scaffold configuration; however, a redshift is observed in the electric quadrupole resonance peak [Figure S3(e)]. This spectral shift indicates a sensitivity to structural or environmental changes, highlighting the system's potential sensing capability. Correspondingly, the Purcell enhancement remains comparable to that of the pillar-without-scaffold structure, with a nearly 5-fold increase in the spontaneous emission rate [Figure S3(f)]. Notably, a redshift in the enhancement peak further supports the presence of a sensing mechanism within the pillar-and-scaffold geometry.

Furthermore, we extend our analysis by exciting the structure with a quantum emitter oriented perpendicular to the xy-plane (i.e., along the z-axis). We first applied this configuration to the dielectric pillar-without-scaffold structure, as shown in [Figure S4(a)]. The resulting scattering efficiency spectrum [Figure S4(b)] exhibited distinct resonances corresponding to electric dipole (ED) and electric quadrupole (EQ) modes, indicating weak coupling between the vertically oriented dipole and the photonic modes of the structure. This interaction led to a moderate Purcell enhancement, with a 3-fold increase in the spontaneous emission rate [Figure S4(c)]. We then perform the same analysis for the pillar-and-scaffold structure, as illustrated in [Figure S4(d)]. The scattering efficiency spectrum revealed a mode pattern similar to that observed in the pillar-without-scaffold configuration. However, both ED and EQ resonance peaks exhibited a redshift [Figure S4(e)]. This spectral shift reflects the structure's sensitivity to geometric or environmental modifications, underscoring its potential for quantum sensing applications. Despite the spectral shift, the Purcell enhancement remained consistent with the pillar-without-scaffold case, exhibiting an approximately 3-fold increase in the spontaneous emission rate [Figure S4(f)]. The observed redshift in both scattering and Purcell spectra further reinforces the role of the scaffolded geometry in enabling environmental responsiveness.

Collectively, these results confirm that variations in the surrounding structural environment induce quantifiable spectral shifts, particularly in higher-order optical resonances. This behavior is indicative of nanoscale quantum sensing functionality, in which subtle changes in the local electromagnetic environment of the system modulate the dynamics of the interaction between emitter and structure.

5.2 Silver-Coated Dielectric Pillar-and-Scaffold Structure

Next, we investigated the impact of a 50 nm thin silver coating on both the pillar-without-scaffold and pillar-and-scaffold dielectric structures, with the quantum emitter positioned 20 nm below the top surface. The position of the dipole is indicated at the top of the schematics, located within the small view of the pillar. The analysis was first performed for the silver-coated pillar-without-scaffold configuration, as shown in [Figure S5(a)], where the structure was excited by a dipole oriented parallel to the x-axis. The corresponding scattering efficiency spectrum [Figure S5(b)] shows sharp resonances associated with magnetic dipole (MD) and electric quadrupole (EQ). This configuration yielded a significant Purcell enhancement, reaching approximately 90-fold [Figure S5(c)]. The substantial increase in the spontaneous emission rate is attributed to strong plasmonic coupling enabled by the metallic coating. Notably, the spectral alignment between the scattering resonances, specifically the dipole and quadrupole modes, and the Purcell enhancement peak confirms that the observed emission rate amplification arises from the efficient coupling of emitter and structural modes due to plasmonic coupling. Subsequently, we performed the same analysis for the silver-coated pillar-and-scaffold structure, as illustrated in [Figure S5(d)]. The structure was excited using a dipole source oriented parallel to the x-axis. The resulting scattering efficiency spectrum, shown in [Figure S5(e)], exhibited modal characteristics similar to those observed in the silver-coated pillar-without-scaffold structure. However, a significantly enhanced Purcell factor of approximately 100-fold was achieved in this case—an order of magnitude greater than the enhancement observed in the pillar-without-scaffold counterpart. This substantial increase is attributed to the effect of the metallic coating and the scaffold geometry, which together facilitate stronger plasmonic coupling and improved confinement of the electromagnetic field around the emitter. These results signify the critical role of structural design in optimizing light–matter interactions for enhanced quantum emission performance.

Lastly, we extended our analysis by exciting the structures using a quantum emitter oriented perpendicular to the xy-plane, i.e., along the z-axis. This configuration was first applied to the silver-coated pillar-without-scaffold structure, as illustrated in [Figure S6(a)]. The corresponding scattering efficiency spectrum [Figure S6(b)], revealed distinct resonances associated with electric quadrupole (EQ) and electric dipole (ED) modes, appearing at wavelengths of 646 nm and 696 nm, respectively. These results confirm effective coupling between the vertically aligned emitter and the supported photonic modes of the structure. The

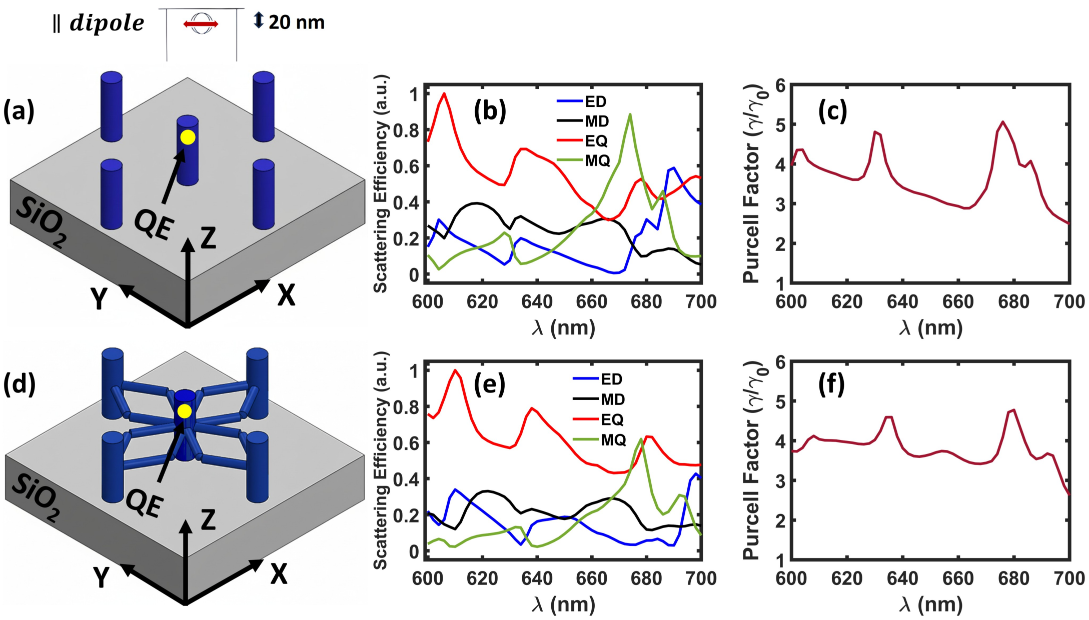

Figure S3. (a) Schematic illustration of a pillar-without-scaffold structure composed of polymer on a silica substrate, excited by a quantum emitter (QE) located 20 nm below the top surface inside the central pillar. The position of the dipole is indicated at the top of the schematics, located within the small view of the pillar. (b–c) Corresponding scattering efficiency (SE) and Purcell factor spectra for the dipole oriented parallel to the x-axis. (d) Schematic illustration of a pillar-and-scaffold structure under the same conditions. (e–f) Corresponding SE and Purcell factor spectra for the dipole oriented parallel to the x-axis.

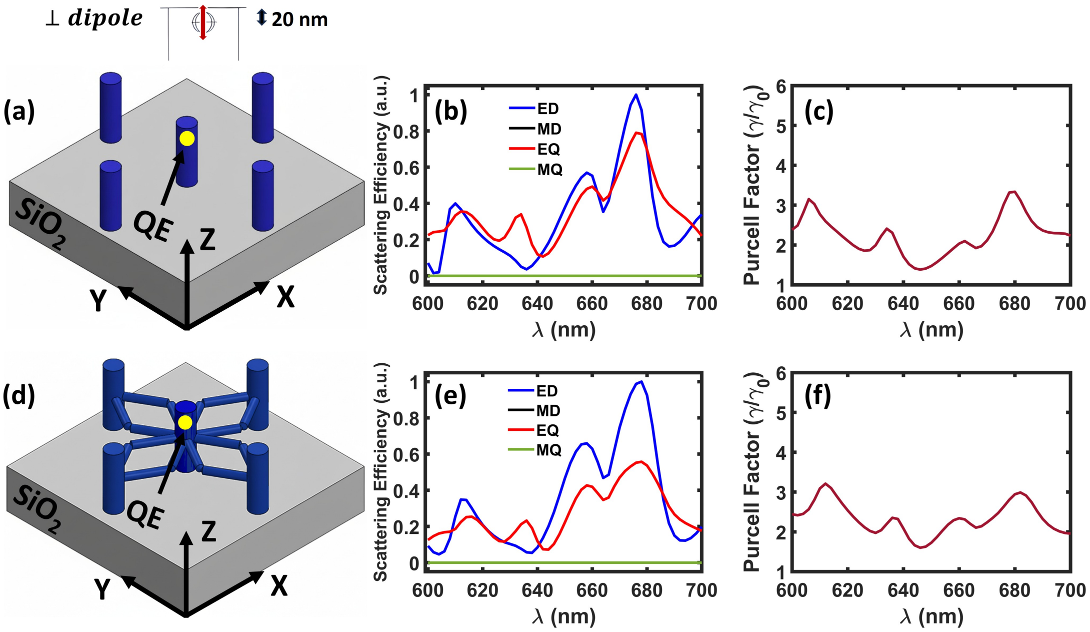

Figure S4. (a) Schematic illustration of a pillar-without-scaffold structure composed of polymer on a silica substrate, excited by a quantum emitter (QE) located 20 nm below the top surface inside the central pillar. The position of the dipole is indicated at the top of the schematics, located within the small view of the pillar. (b–c) Corresponding scattering efficiency (SE) and Purcell factor spectra for the QE oriented perpendicular to the xy-plane (i.e., along the z-axis). (d) Schematic illustration of a pillar-and-scaffold structure under the same conditions. (e–f) Corresponding SE and Purcell factor spectra for the QE oriented perpendicular to the xy-plane.

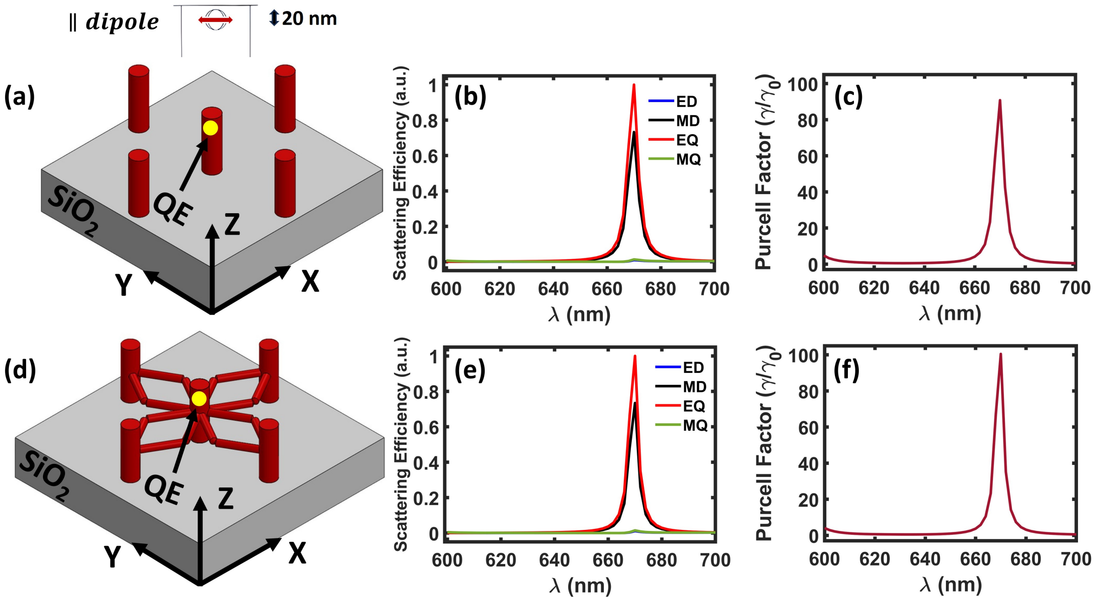

Figure S5. (a) Schematic illustration of a silver-coated pillar-without-scaffold structure composed of polymer on a silica substrate, excited by a quantum emitter (QE) located 20 nm below the top surface inside the central pillar. The position of the dipole is indicated at the top of the schematics, located within the small view of the pillar. (b–c) Corresponding scattering efficiency (SE) and Purcell factor spectra for the QE oriented parallel to the x-axis. (d) Schematic illustration of a silver-coated pillar-and-scaffold structure under the same conditions. (e–f) Corresponding SE and Purcell factor spectra for the QE oriented parallel to the x-axis.

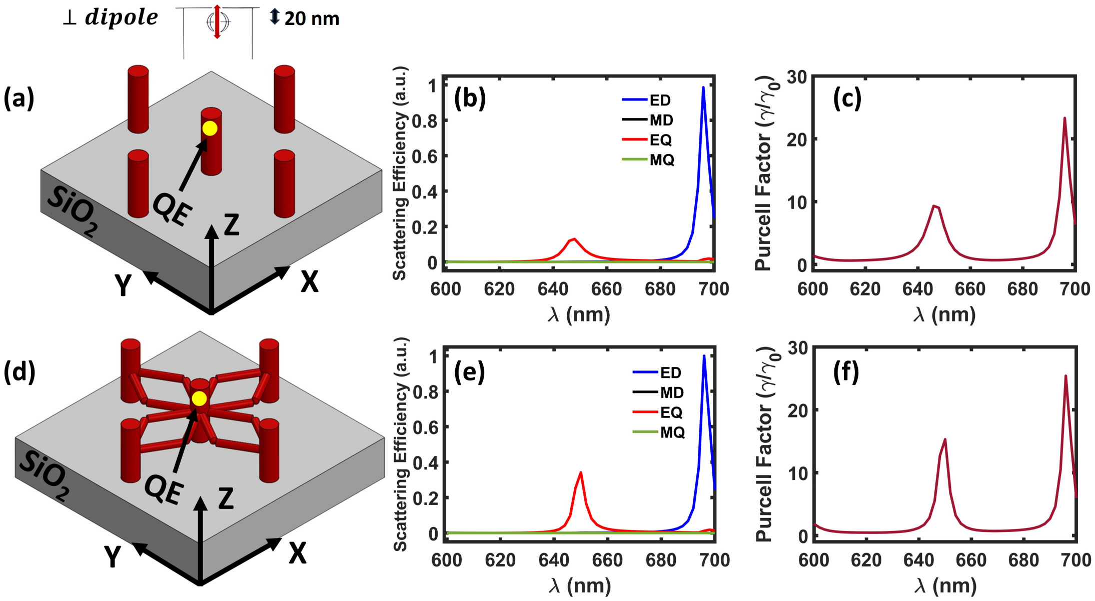

Figure S6. (a) Schematic illustration of a silver-coated pillar-without-scaffold structure composed of polymer on a silica substrate, excited by a quantum emitter (QE) located 20 nm below the top surface inside the central pillar. (b–c) Corresponding scattering efficiency (SE) and Purcell factor spectra for the QE oriented perpendicular to the xy-plane. (d) Schematic illustration of a silver-coated pillar-and-scaffold structure under the same conditions. (e–f) Corresponding SE and Purcell factor spectra for the QE oriented perpendicular to the xy-plane.

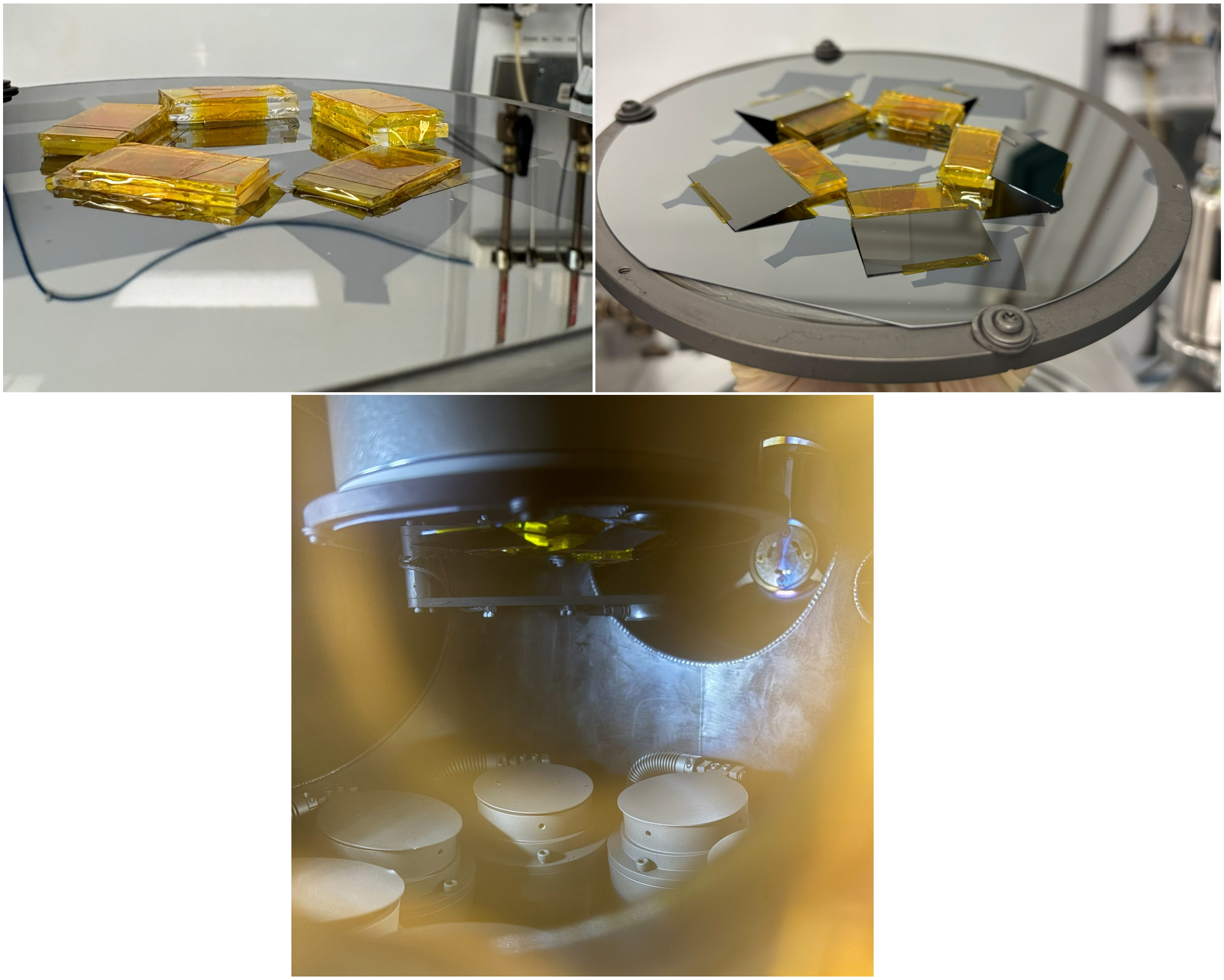

Figure S7. Experimental setup showing the use of plastic shims to achieve angled Ag deposition (10°, 25°, and 45°) for uniform coating of the 3-D scaffold structures during Orion magnetron DC sputtering.

Purcell enhancement spectrum [Figure S6(c)] exhibits notable increases in the spontaneous emission rate, with enhancements of approximately 9-fold and 23-fold at the EQ and ED resonance wavelengths, respectively. These enhancements further highlight the interaction of the emitter with different multipolar modes supported by the silver-coated structure. We further extended this analysis to the silver-coated pillar-and-scaffold structure [Figure S6(d)]. The corresponding scattering efficiency spectrum [Figure S6(e)], exhibited similar modal behavior to that of the silver-coated pillar-without-scaffold structure, with clearly identifiable EQ and ED resonances. However, the resonance amplitudes were noticeably higher in the scaffolded configuration, indicating stronger mode excitation. This enhancement in modal strength was also reflected in the Purcell factor spectrum, shown in [Figure S6(f)], where emission rate enhancements of approximately 15-fold and 26-fold were observed at the EQ and ED resonance wavelengths, respectively. These results demonstrate the influence of the scaffold geometry in further boosting light–matter interactions, likely due to enhanced plasmonic confinement and improved field overlap with the quantum emitter.

References

1. Y. Wang, H. Liu *et al.*, *Journal of Applied Physics*, 2013, **113**, 013503.
2. M. A. Green, *Solar Energy Materials and Solar Cells*, 2008, **92**, 1305–1310.
3. P. B. Johnson and R. W. Christy, *Physical Review B*, 1972, **6**, 4370–4379.
4. H. R. Phillip and E. A. Taft, *Physical Review*, 1964, **136**, A1445.
5. M. A. Ahamad, N. Ahmed, S. Castelletto and F. A. Inam, *Journal of Lightwave Technology*, 2023, **42**, 689–695.
6. F. Inam, T. Gaebel, C. Bradac, L. Stewart, M. Withford, J. Dawes, J. Rabeau and M. Steel, *New Journal of Physics*, 2011, **13**, 073012.
7. F. A. Inam and S. Castelletto, *Journal of the Optical Society of America B*, 2021, **38**, 3697–3704.
8. M. H. A. K. Khushik and C. Jiang, *Results in Physics*, 2019, **13**, 102138.
9. W. L. Barnes, S. A. Horsley and W. L. Vos, *Journal of Optics*, 2020, **22**, 073501.
10. S. Haroche and J.-M. Raimond, *Exploring the quantum: atoms, cavities, and photons*, Oxford university press, 2006.
11. P. Lodahl and S. Stobbe, *Nanophotonics*, 2013, **2**, 39–55.
12. N. Gisin, G. Ribordy, W. Tittel and H. Zbinden, *Reviews of modern physics*, 2002, **74**, 145.
13. L. Novotny and B. Hecht, *Principles of nano-optics*, Cambridge university press, 2012.
14. R. Carminati and M. Gurioli, *Optics Express*, 2022, **30**, 16174–16183.
